# Loss of mitochondrial amidoxime-reducing component 1 (mARC1) prevents disease progression by reducing fibrosis in multiple mouse models of chronic liver disease

**DOI:** 10.1097/HC9.0000000000000637

**Published:** 2025-02-10

**Authors:** Erin S. Coyne, Yilin Nie, Darwin Lee, Sentibel Pandovski, Tiffany Yang, Heather Zhou, Thomas W. Rosahl, Ester Carballo-Jane, Desiree Abdurrachim, Yongqi Zhou, Christopher Hendra, Asad Abu Bakar Ali, Stacey Meyers, Wendy Blumenschein, Brendan Gongol, Yang Liu, Yingjiang Zhou, Saswata Talukdar

**Affiliations:** 1 Department of Cardiometabolic Disease, Merck & Co. Inc., South San Francisco, California, USA; 2 Department of Quantitative Biosciences, Merck & Co. Inc., Rahway, New Jersey, USA; 3 Department of Data, AI & Genome Sciences, MSD, Singapore

**Keywords:** MASLD, MASH, mARC, fibrosis, lipotoxicity

## Abstract

**Background:**

Metabolic dysfunction–associated steatotic liver disease is a prevalent disease that affects nearly one-third of the global population. Recent genome-wide association studies revealed that a common missense variant in the gene encoding mitochondrial amidoxime reducing component 1 (mARC1) is associated with protection from metabolic dysfunction–associated steatotic liver disease, all-cause cirrhosis, and liver-related mortality suggesting a role for mARC1 in liver pathophysiology; however, little is known about its function in the liver. In this study, we aimed to evaluate the impact of mARC1 hepatoprotective variants on protein function, the effect of loss of mARC1 on cellular lipotoxic stress response, and the effect of global or hepatocyte-specific loss of mARC1 in various mouse models of metabolic dysfunction–associated steatohepatitis and liver fibrosis.

**Methods﻿ and Results:**

Expression and characterization of mARC1 hepatoprotective variants in cells and mouse liver revealed that the mARC1 p.A165T exhibited lower protein levels but maintained its mitochondrial localization. In cells, the knockdown of mARC1 improved cellular bioenergetics and decreased mitochondrial superoxide production in response to lipotoxic stress. Global genetic deletion and hepatocyte-specific knockdown of mARC1 in mice significantly reduced liver steatosis and fibrosis in multiple mouse models of metabolic dysfunction–associated steatohepatitis and liver fibrosis. Furthermore, RNA-seq analysis revealed that the pathways involved in extracellular matrix remodeling and collagen formation were downregulated in the liver, and the plasma lipidome was significantly altered in response to the loss of mARC1 in mice.

**Conclusions:**

Overall, we have demonstrated that loss of mARC1 alters hepatocyte response to lipotoxic stress and protects mice from diet-induced MASH and liver fibrosis consistent with findings from human genetics.

## INTRODUCTION

Metabolic dysfunction–associated steatotic liver disease (MASLD) is estimated to affect more than one-third of the global population,^1^,^2^ and its prevalence is increasing in parallel with increases in metabolic syndrome, obesity, and type 2 diabetes in many countries.^2^ MASLD can progress to more severe forms of disease, including metabolic dysfunction–associated steatohepatitis (MASH), cirrhosis, HCC, and ultimately, death.^3^,^4^,^5^,^6^,^7^,^8^ Disease progression, particularly the development of liver fibrosis, significantly increases the risk of liver-related mortality.^6^,^7^,^9^,^10^ Although the recent FDA approval of the thyroid hormone beta receptor agonist, resmetirom, for the treatment of MASH is a much-needed therapeutic for patients,^11^ novel therapies targeting independent and complementary pathways that can reduce liver fibrosis are still needed.

Recent genome-wide association studies have identified several human genes with potential causal relationships to chronic liver disease phenotypes, including mitochondrial amidoxime-reducing component 1 (*MTARC1*, encoding the protein mARC1). A common missense variant within the *MTARC1* gene p.A165T, is associated with decreased liver fat, lower ALT, lower total cholesterol, protection from all-cause cirrhosis, and reduction in liver-related mortality.^12^,^13^,^14^ Furthermore, the rare missense variant p.M187K and loss of function variants p.R200Ter and p.R305Ter, are also associated with liver protective phenotypes in humans,^12^,^15^ suggesting that mARC1 may play a critical role in the pathogenesis of liver disease.

mARC1 is a molybdenum-cofactor–dependent enzyme and a principal component of the mitochondrial N-reductive system comprised of cytochrome b5 reductase, cytochrome b5, and mARC.^16^,^17^ It is localized to the outer mitochondrial membrane^18^ where it catalyzes the reduction of N-oxygenated substrates.^19^,^20^,^21^,^22^,^23^,^24^,^25^,^26^,^27^ While the enzymatic activity of mARC1 has been well-elucidated^16^ and biochemical substrates, including metabolites such as N-omega-hydroxy-L-arginine, hydroxylated nucleic bases, and certain prodrugs with amidoxime functional groups have been identified,^19^,^24^,^25^ very little is known about the role of mARC1 in physiology. Indeed, the endogenous substrate(s) that confers mARC1 function and may be causal for disease progression remains elusive. mARC1 has a closely related paralog, mitochondrial amidoxime-reducing component 2 (mARC2), that has similar substrate specificity,^19^ and both mARC1 and mARC2 are expressed in the liver. However, there are no reports of human genetic associations between variants in the *MTARC2* gene and liver disease suggesting that *MTARC1* may be the critical mARC enzyme involved in liver pathophysiology.

Several recent reports have highlighted the role of mARC1 in rodent models of liver disease, demonstrating that the loss of mARC1 by siRNA knockdown can reduce liver triglycerides and decrease circulating ALT and cholesterol levels^28^,^29^,^30^ which is consistent with human genetics. In contrast, a recent report described, for the first time, the phenotype of an *Mtarc1* knockout (KO) mouse and found no effect of the loss of mARC1 on liver phenotypes in response to a variety of MASH-inducing diets.^31^ In this study, to resolve these discrepancies, we sought to characterize the function of several mARC1 human genetic variants and confirm that the loss of function of mARC1 recapitulates the hepatoprotective phenotype observed in humans. We observed that expression of mARC1 p.A165T variant in cells and mouse liver results in lower steady-state protein levels but does not disrupt mitochondrial localization and that knockdown of *MTARC1* protects cells from lipotoxic stress and improves mitochondrial bioenergetics. In several mouse models of MASH and liver fibrosis, we found that loss of mARC1 by genetic deletion or siRNA-mediated hepatocyte-specific knockdown resulted in improved liver endpoints including decreased steatosis, fibrosis, and circulating ALT and total cholesterol levels. Together, our results highlight the critical role that mARC1 plays in liver pathogenesis and furthers our understanding of mARC1’s biological function. With the translation of preclinical models toward the pharmacological modulation of mARC1 in mind, the studies reported here will help inform the selection of appropriate pharmacological modalities and refine the selection of patient populations in the clinic.

## METHODS

### Animal studies

Animal experiments were conducted in accordance with the Public Health Service Policy on Humane Care and Use of Laboratory Animals from the Office of Laboratory Animal Welfare, and the Guide for the Care and Use of Laboratory Animals from the National Research Council. All experiments were approved by the Institutional Animal Care and Use Committee of Merck & Co. Inc., South San Francisco, CA, USA, and were performed in accordance with the relevant guidelines and regulations at MRL South San Francisco. All mice were housed in a temperature- and humidity-controlled environment with a 12-hour light-dark cycle, environmental enrichment, and ad libitum access to food and water. Male C57BL/6J mice from Jackson Laboratory were used for siRNA studies and viral vector expression studies and male C57BL/6NTac *Mtarc1* KO mice were used for genetic studies.

### *Mtarc1* KO mouse generation

*Mtarc1* KO mice were generated at Taconic Biosciences Inc. for Merck & Co. Inc., Rahway, NJ (Mosc1 [Marc1]—Model 10489—KO | Taconic Biosciences). Briefly, exons 3 to 6 of *Mtarc1* were flanked by loxP sites by homologous recombination in embryonic stem cells. The constitutive KO allele was generated after Cre-mediated recombination by crossing the chimera with a Cre-Deleter on the C57BL/6 genetic background. Deletion of exons 3 to 6 results in loss of function of mARC1 by deleting part of the MOSC domain and by generating a frameshift from exon 2 to exon 7 with a premature stop codon in exon 7.

### Statistical analysis

All data were expressed as mean +/− SEM. Statistical analysis was performed with GraphPad Prism 9 (GraphPad Software). For single comparisons, Student *t* test was performed. For multiple comparisons, one-way or two-way ANOVA with Dunnett multiple comparison tests were used. For nonparametric analyses, Kruskal-Wallis and Dunn multiple comparison tests were performed. Differences are marked as *p<0.05, **p<0.01, and ***p<0.001, ****p<0.0001. The detailed methodology can be found in the Supplemental Methods, http://links.lww.com/HC9/B878.

## RESULTS

### Hepatoprotective mARC1 A165T variant exhibits lower protein abundance but still localizes to mitochondria in Huh7 cells

**FIGURE 1:**
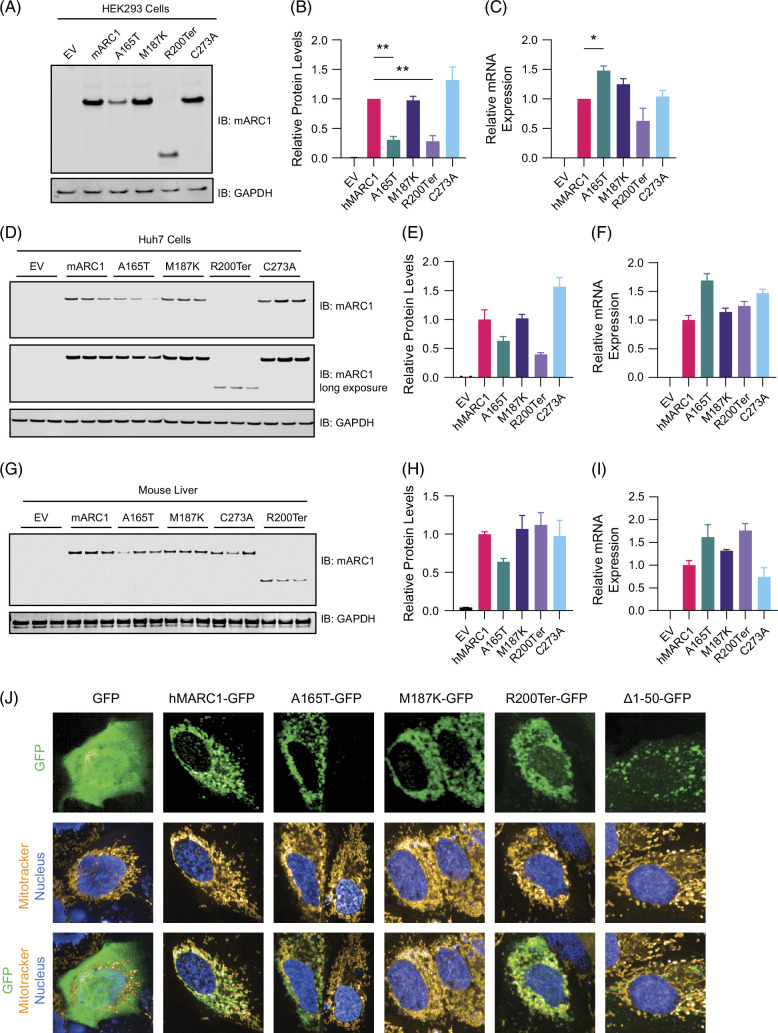
Hepatoprotective mARC1 A165T variant exhibits lower protein abundance but still localizes to mitochondria in Huh7 cells. (A–C) Equal amounts of human mARC1 expression plasmids were transiently transfected into HEK293 cells and cells were harvested after 48 hours for qPCR and immunoblot analysis. (A) Immunoblot for mARC1 and GAPDH. (B) Quantification by densitometry of immunoblot from (A). (C) qPCR analysis of human mARC1 mRNA expression. (D–F) Equal amounts of human mARC1 expression plasmids were transiently transfected into Huh7 cells and cells were harvested after 48 hours for qPCR and immunoblot analysis. (D) Immunoblot for mARC1 and GAPDH. Long exposure of blot presented to reveal bands that were not observed with short exposure. (E) Quantification by densitometry of immunoblot from (A). (C) qPCR analysis of human mARC1 mRNA expression. (G–I) AAV8 expressing human mARC1 variants was delivered to mouse liver by intravenous injection. Eleven days later, livers were collected for qPCR and immunoblot analysis. (G) Immunoblot for mARC1 and GAPDH. (H) Quantification by densitometry of immunoblot in (G). (I) qPCR analysis of human mARC1 mRNA expression. (J) GFP-tagged mARC1 expression plasmids were transiently transfected into Huh7 cells. 24 hours later cells were incubated with Mitotracker dye to label mitochondria and DAPI to label nuclei. Representative images were captured on Opera Phenix High Content Screening system with 63x objective. Data are presented as mean +/− SEM. n=3 *p<0.05, **p<0.01. Abbreviations: EV, empty vector﻿; GFP, green fluorescent protein﻿; mARC1, mitochondrial amidoxime reducing component 1.

We sought to evaluate the effect of the mARC1 hepatoprotective variants p.A165T, p.M187K, p.R200Ter, and mARC1 catalytically inactive C273A mutation on mARC1 protein function. Previously published in silico analysis suggested that the A165T variant may lead to protein instability due to the mutation occurring in a critical alpha helix^32^; however, structural analysis of mARC1 A165T variant using X-ray crystallography revealed a nearly identical structure to the mARC1 risk variant demonstrating that impact of this variant on protein structure, stability, and function is not clear. Additional recent reports have revealed that the A165T variant has lower protein abundance due to more rapid protein degradation.^31^,^33^,^34^ First, we expressed these variants in HEK293 cells and observed that the A165T and R200Ter variants had significantly decreased protein levels (Figure 1A, B) but increased mRNA levels (Figure 1C). Next, we expressed these variants in a well-characterized Huh7 hepatocellular carcinoma cell line. Expression of these mARC1 mutant proteins in Huh7 cells revealed that the protective A165T and R200Ter variants resulted in decreased steady-state levels of protein (Figure 1D, E) while the protective M187K variant and the catalytically inactive C273A mutant did not result in a significant change in protein levels (Figure 1D, E) consistent with what was observed in HEK293 cells. In addition, we consistently observed that overexpression of the A165T variant resulted in significantly elevated RNA levels compared with the other variants (Figure 1F). Next, we expressed these hepatoprotective human mARC1 variants in mouse livers, to understand how the RNA and protein levels of these variants are regulated in a more physiological context. In the mouse liver, we observed a decrease in A165T protein levels, but not M187K, R200Ter, or C273A protein levels (Figure 1G-H). Consistent with our previous observation in cells, we also observed a significant increase in mARC1 A165T mRNA levels (Figure 1I) concomitant with a decreased protein level, suggesting that post-transcriptional regulation of this variant may be altered. Next, we investigated whether these variants of mARC1 could alter its subcellular localization. mARC1 has an N-terminal mitochondrial targeting signal that results in its localization to the outer mitochondrial membrane in an N-in, C-out orientation.^18^ Recent reports have suggested that the mARC1 A165T variant may have decreased mitochondrial localization owing to more rapid degradation of the protein.^31^,^35^ We tested whether C-terminal green fluorescent protein-tagged mARC1 mutants (A165T, M187K, R200Ter, and C273A) would colocalize with a MitoTracker dye in Huh7 cells transiently transfected with the green fluorescent protein expression constructs. We found that green fluorescent protein-tagged mARC1 variants A165T, M187K, and R200Ter all expressed well in cells (Supplemental Figure S1, http://links.lww.com/HC9/B878) and co-localized with MitoTracker dye but a mARC1 expression construct lacking amino acids 1-50 (mitochondrial targeting signal) did not (Figure 1J), suggesting that these protective variants have a limited impact on mARC1 mitochondrial localization in Huh7 cells.

### Loss of mARC1 and mARC2 protects cells from lipotoxicity

**FIGURE 2:**
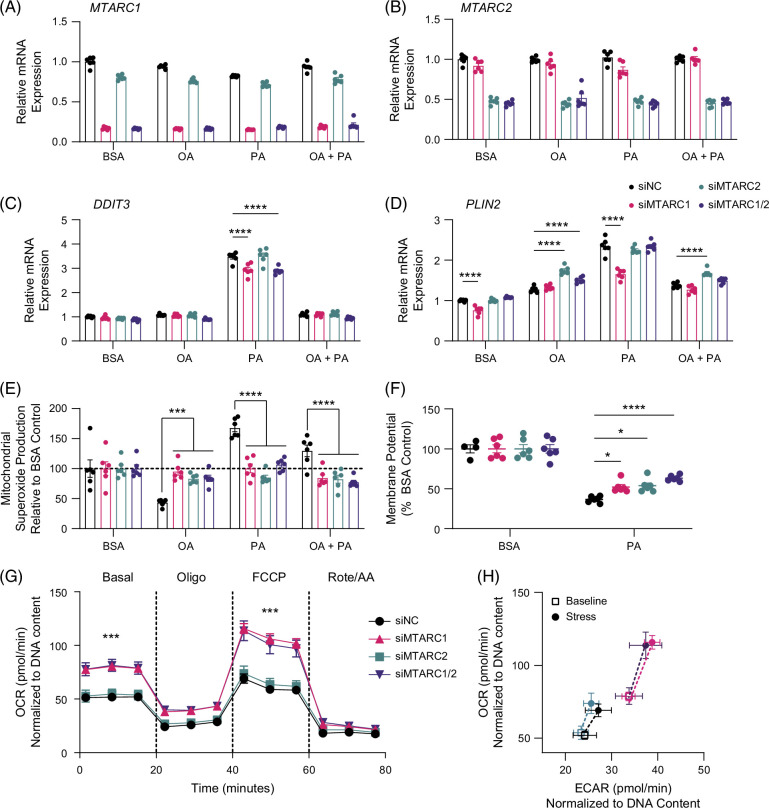
Knockdown of mARC1 and mARC2 protects cells from hepatotoxicity. mARC1 and/or mARC2 were knocked down in Huh7 cells and treated with 400 μM BSA-oleate (OA) and/or BSA-palmitate (PA) or BSA control. (A–D) mRNA expression of *MTARC1, MTARC2, DDIT3, and PLIN2*. (E) Mitochondrial superoxide levels normalized to BSA control. (F) Mitochondrial membrane potential normalized to BSA control. (G) Oxygen consumption during the mitochondrial stress test measured using Seahorse XF Pro Metabolic Analyzer after knockdown of *MTARC1* and/or *MTARC2* in Huh7 cells. n=7–19 per well. (H) Cellular energy phenotype comparing shift in OCR and ECAR from baseline to stressed state during mitochondrial stress test. Data are presented as mean +/−​​​​​​ SEM. n=6 *p<0.05, ***p<0.001, ****p<0.0001. Abbreviations: BSA, bovine serum albumin; ECAR, extracellular acidification rate; FCCP, carbonyl cyanide p-(trifluoromethoxy)phenylhydrazone; MTARC1, mitochondrial amidoxime reducing component 1; OA, oleate; OCR, oxygen consumption rate; PA, palmitate.

Based on our findings that the mARC1 A165T variant is less abundant at the protein level and to validate the human genetic findings suggesting that loss of mARC1 function is associated with protection from liver fat accumulation,^12^,^13^ we knocked down mARC1 and/or mARC2 by siRNA in cells and challenged the cells with established lipotoxic stressors palmitic acid and/or oleic acid. *MTARC1* was robustly knocked down by over 80%, whereas *MTARC2* was knocked down by over 50% (Figure 2A, B). We observed significant upregulation of several genes in response to palmitate-induced lipotoxic stress, including *DDIT3*, *PLIN2*, *PPARGC1A*, and *CPT1A* (Figure 2C, D and Supplemental Figure S2, http://links.lww.com/HC9/B878). Knockdown of *MTARC1* significantly reduced the upregulation of DDIT3 and PLIN2 in response to palmitate stress, whereas knockdown of *MTARC2* only reduced the upregulation of DDIT3 (Figure 2C, D). In addition, we observed a significant decrease in genes involved in oxidative stress *NRF1*, *NFE2L2*, and *SOD1* when *MTARC1* was knocked down both in the presence and absence of lipotoxic stress (Supplemental Figure S2A–C, http://links.lww.com/HC9/B878). Knockdown of *MTARC1* and *MTARC2* significantly reduced mitochondrial superoxide production and blunted the reduction in mitochondrial membrane potential in response to palmitate-induced stress (Figure 2E, F), suggesting that loss of mARC1 and/or mARC2 can protect mitochondria from lipotoxic stress. To further evaluate the impact of the loss of mARC1 and mARC2 on mitochondrial function, we evaluated cellular bioenergetics and observed that knockdown of *MTARC1*, but not *MTARC2*, increased basal and maximal oxygen consumption rates and shifted the cells toward a more energetic phenotype (increased oxygen consumption rates and extracellular acidification rates) in response to the mitochondrial stress test (Figure 2G, H), suggesting that loss of mARC1 can protect mitochondria from lipotoxic stress and improve cellular bioenergetics.

### Mtarc1 gene deletion results in decreased liver fibrosis in male mice fed a high-fat, high-fructose, high-cholesterol diet

**FIGURE 3:**
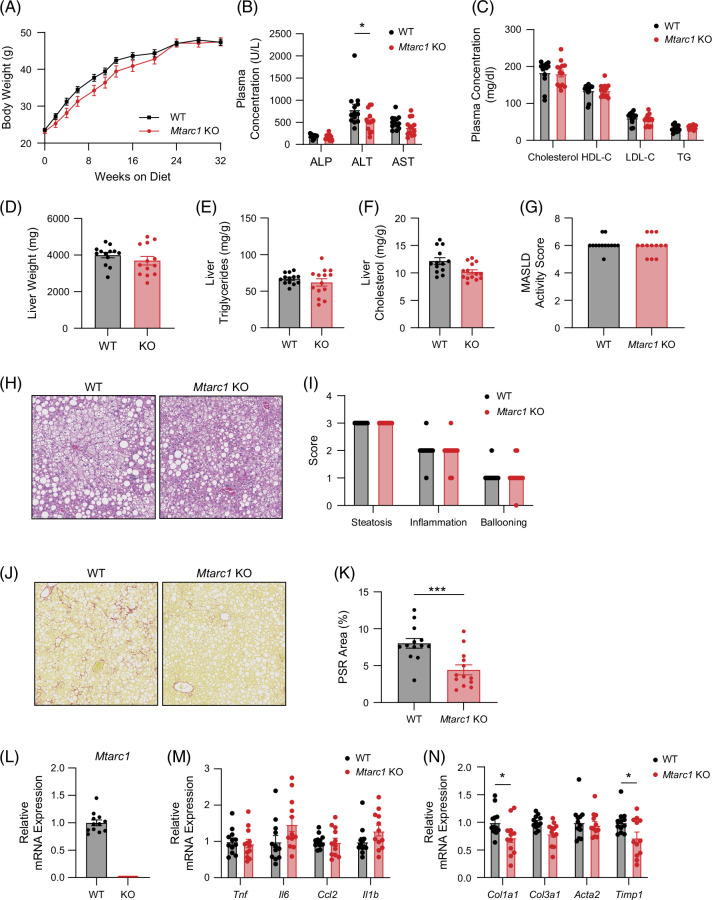
*Mtarc1* gene deletion results in decreased liver fibrosis in male mice fed a high-fat, high-fructose, high-cholesterol diet (GAN diet). Male WT and *Mtarc1* KO mice were fed a GAN diet to induce MASH and liver fibrosis for 32 weeks. (A) Body weight growth curves over 32 weeks on the GAN diet. (B) Plasma liver enzyme levels at 32-week time point. (C) Plasma lipid levels at the 32-week time point. (D) Liver weight, (E) liver triglycerides, and (F) liver cholesterol at the 32-week time point. (G) Pathologist assessed MASLD activity score. (H) Representative H&E images of liver from WT and *Mtarc1* KO mice at the 32-week time point. (I) Pathologist assessed steatosis, inflammation, and ballooning score. (J) Representative Picrosirius red (PSR) stained images of liver WT and *Mtarc1* KO mice at the 32-week time point. (K) Quantification of the PSR stained area within the whole liver lobe. (L) mRNA expression of *Mtarc1* in the liver at the 32-week time point. (M) mRNA expression of inflammatory genes at the 32-week time point. (N) mRNA expression of fibrosis-associated genes at the 32-week time point. Data are presented as mean +/− SEM. n=13–14 *p<0.05, ***p<0.001. Abbreviations: KO, knockout; *MTARC1*, mitochondrial amidoxime reducing component 1; PSR, picrosirius red; TG, triglyceride; WT, wild type.

We generated and characterized *Mtarc1* KO mice (Supplemental Information, http://links.lww.com/HC9/B878 and Supplemental Figure S3, http://links.lww.com/HC9/B878). To evaluate whether genetic deletion of *Mtarc1* in mice results in hepatoprotective effects, we challenged wild-type and *Mtarc1* KO mice with a high-fat, high-fructose, high-cholesterol diet also known as the Gubra Amylin NASH (GAN) diet, for 32 weeks starting at 8–10 weeks of age. As expected, mice fed the GAN diet gained a significant amount of weight, but there was no significant difference in body weight at the 32-week time point between genotypes (Figure 3A). There was a significant decrease in plasma ALT levels in *Mtarc1* KO mice, but no differences were observed in plasma lipids between genotypes (Figure 3B, C). In addition, there were no significant differences in liver weight (Figure 3D), liver triglycerides (Figure 3E), or liver cholesterol levels (Figure 3F) between the genotypes. Histological analysis and scoring of MASLD activity score revealed no significant difference between genotypes in steatosis, inflammation, and ballooning scores (Figure 3G–I) but analysis of liver fibrosis by picrosirius red staining revealed a nearly 50% reduction in liver fibrosis in *Mtarc1* KO mice (Figure 3J, K). Analysis of mRNA levels revealed that *Mtarc1* mRNA was not detectable in *Mtarc1* KO mice (Figure 3L), there were no significant differences in inflammatory gene expression between genotypes (Figure 3M), and *Col1a1* and *Timp1* mRNA expression was significantly decreased in *Mtarc1* KO mice (Figure 3N) consistent with decreased liver fibrosis.

### Mtarc1 gene deletion results in decreased liver triglyceride accumulation and liver fibrosis in male mice fed a highly lipogenic high-fat diet supplemented with fructose water

**FIGURE 4:**
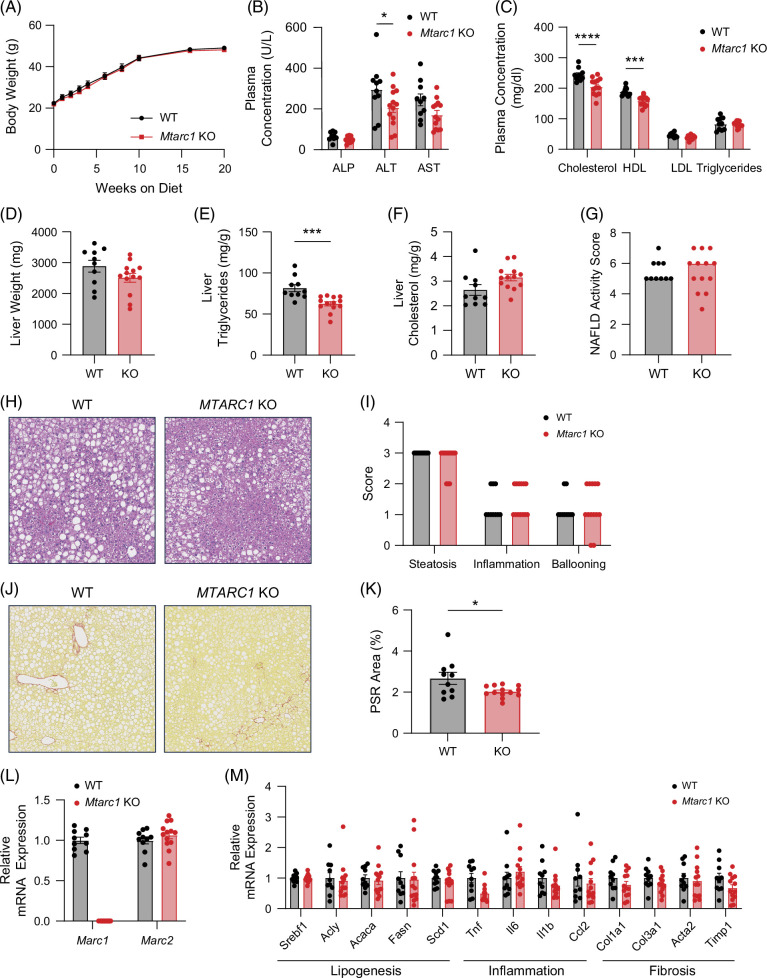
*Mtarc1* gene deletion results in decreased liver triglyceride accumulation and decreased liver fibrosis in male mice fed a high-fat diet supplemented with fructose water. Male WT and *Mtarc1* KO mice were fed an HFD supplemented with 30% w/v fructose water (HFDHFr) for 20 weeks to induce MASH and liver fibrosis. (A) Body weight growth curves over 20 weeks on HFDHFr. (B) Plasma liver enzyme levels and (C) plasma lipid levels at the 20-week time point. (D) Liver weight, (E) liver triglycerides, and (F) liver cholesterol levels. (G) Pathologist assessed MASLD activity score. (H) Representative H&E images of liver from WT and *Mtarc1* KO mice at the 20-week time point. (I) Pathologist assessed steatosis, inflammation, and ballooning scores. (J) Representative Picrosirius red (PSR) stained images of liver WT and *Mtarc1* KO mice at the 20-week time point. (K) Quantification of the PSR stained area within the whole liver lobe. (L) mRNA expression of *Mtarc1* and *Mtarc2* in liver. (M) Liver mRNA expression of lipogenic, inflammatory, and fibrosis-associated genes. Data are presented as mean +/− SEM. n=10–13 *p<0.05, ***p<0.001, ****p<0.0001. Abbreviations: KO, knockout; *MTARC1*, mitochondrial amidoxime reducing component 1; PSR, picrosirius red; TG, triglyceride; WT, wild type.

Since human genetics suggests that hepatoprotective variants within the *MTARC1* gene will also affect liver fat content, we challenged wild-type and *Mtarc1* KO mice with a highly lipogenic MASH diet model (high-fat diet supplemented with fructose water—HFDHFr) for 20 weeks to assess this endpoint. In this diet model, body weights increased significantly over the course of 20 weeks but were similar between genotypes (Figure 4A). There was a significant decrease in plasma ALT (Figure 4B), and a significant decrease in plasma total and HDL cholesterol (Figure 4C) in *Mtarc1* KO mice. In addition, there were no significant differences in liver weight (Figure 4D) or liver cholesterol levels (Figure 4F); however, there was a significant decrease in liver triglycerides in *Mtarc1* KO mice (Figure 4E). Histological analysis of MASLD activity score revealed no significant differences in steatosis, inflammation, or ballooning scores between genotypes (Figure 4G–I). However, a significant 24% decrease in the Picrosirius red area was observed in *Mtarc1* KO mice (Figure 4J-K). *Mtarc1* was not detected at the mRNA level (Figure 4L), and no significant changes in lipogenesis, inflammation, or fibrosis-associated gene expression were observed in *Mtarc1* KO livers (Figure 4M). Taken together, this data supports the hypothesis that loss of mARC1 can reduce liver triglyceride accumulation and decrease liver fibrosis in a highly lipogenic diet model.

We further evaluated the effect of *Mtarc1* gene deletion on liver fibrosis in choline-deficient amino acid-defined high-fat diet and CCl4-induced liver injury models and found a significant decrease in liver fibrosis in both models (Supplemental Figure S4 and S5, http://links.lww.com/HC9/B878).

### Hepatocyte-specific Mtarc1 knockdown results in decreased liver fibrosis in male mice fed a high-fat, high-fructose, high-cholesterol diet

**FIGURE 5:**
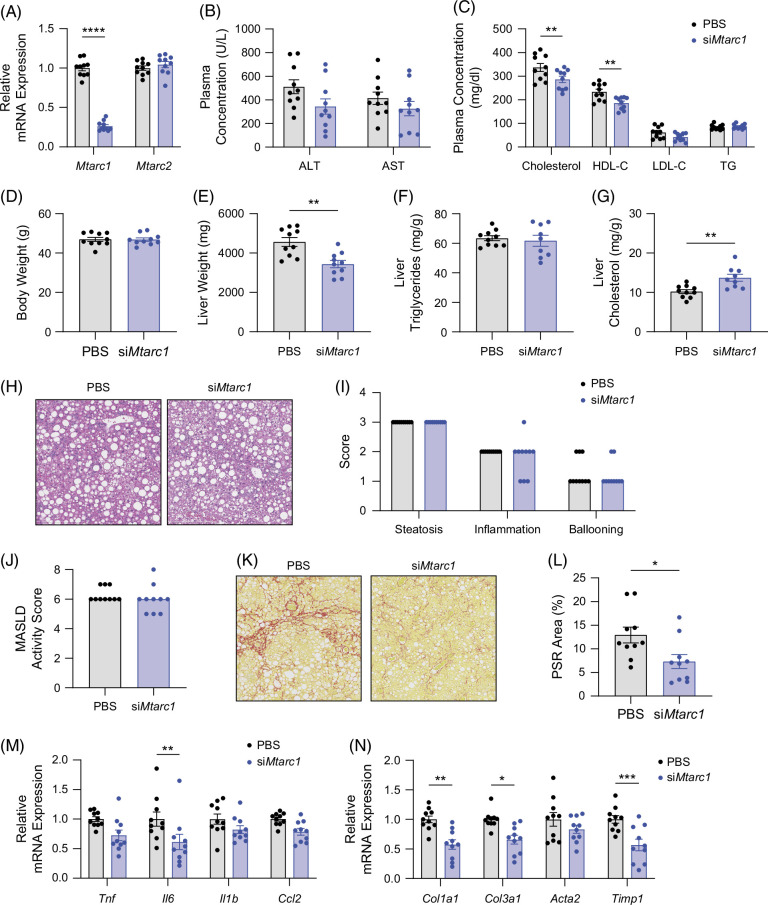
Hepatocyte-specific *Mtarc1* knockdown results in decreased liver fibrosis in male mice fed a GAN diet. Male C57BL/6J mice were fed GAN diet for 16 weeks to induce MASH and liver fibrosis and then treated with GalNAc-si*Mtarc1* or PBS Q2W for an additional 8 weeks. (A) mRNA expression of *Mtarc1* and *Mtarc2* in liver. (B) Plasma liver enzyme levels and (C) plasma lipid levels at the 24-week time point. (D) Body weight after 8 weeks of siRNA treatment and 24 weeks on diet (E) Liver weight, (F) liver triglycerides, and (G) liver cholesterol levels. (H) Representative H&E images of liver from PBS and si*Mtarc1* treated mice. (I) The pathologist assessed steatosis, inflammation, and ballooning scores. (J) Pathologist assessed MASLD activity score. (K) Representative Picrosirius red (PSR) stained images of liver PBS and si*Mtarc1* treated mice. (L) Quantification of the PSR stained area within the whole liver lobe. (M) Liver mRNA expression of inflammatory genes. (N) Liver mRNA expression of fibrosis-associated genes. Data are presented as mean +/− SEM. n=10–13 *p<0.05, **p<0.01, ***p<0.001, ****p<0.0001. Abbreviations: *MTARC1*, mitochondrial amidoxime reducing component 1; PSR, picrosirius red; TG, triglyceride.

To understand whether liver-specific loss of mARC1 can also confer protection from the development of MASH and liver fibrosis, we used a GalNAc-conjugated hepatocyte-targeting siRNA to knockdown *Mtarc1* specifically in hepatocytes in a therapeutic paradigm in mice fed a GAN diet for 16 weeks. After 8 weeks of biweekly siRNA treatment, *Mtarc1* was knocked down by 75% at the RNA level in the liver with no concomitant upregulation of *Mtarc2* RNA levels (Figure 5A). We observed no significant differences in the plasma levels of liver function enzymes ALT or AST (Figure 5B), but there was a significant decrease in total and HDL cholesterol when *Mtarc1* was knocked down in hepatocytes (Figure 5C). Consistent with the genetic KO of *Mtarc1*, there was no significant effect of *Mtarc1* knockdown on body weight (Figure 5D), but there was a significant decrease in liver weight (Figure 5E). *Mtarc1* knockdown did not affect liver triglyceride levels (Figure 5F), but significantly increased liver cholesterol levels (Figure 5G). Histological analysis of hematoxylin and eosin–stained liver sections showed no significant differences in liver steatosis, inflammation, ballooning, or overall MASLD activity scores when *Mtarc1* was knocked down (Figure 5H–J) but further histological evaluation of liver fibrosis revealed a significant decrease in liver collagen in *Mtarc1* siRNA-treated mice (Figure 5K, L) and gene expression evaluation by quantitative polymerase chain reaction showed a significant decrease in inflammation and fibrosis-associated gene expression (Figure 5M, N).

We further evaluated the impact of *Mtarc1* knockdown on liver fibrosis at a later time point on the GAN diet (24 wk followed by 8 weeks of biweekly siRNA treatment) with higher disease burden and found no significant effect on liver endpoints (Supplemental Information, http://links.lww.com/HC9/B878 and Supplemental Figure S6, http://links.lww.com/HC9/B878). We also evaluated the impact of hepatocyte-specific knockdown in the choline-deficient amino acid-defined high-fat diet model and did not observe an impact of the loss of mARC1 on liver fibrosis (Supplemental Figure S8, http://links.lww.com/HC9/B878). Taken together, these data suggest that disease burden at the timing of intervention is critical to fibrosis prevention due to loss of mARC1.

### Hepatocyte-specific Mtarc1 knockdown results in decreased liver steatosis and fibrosis in male mice fed a high-fat diet supplemented with fructose water

**FIGURE 6:**
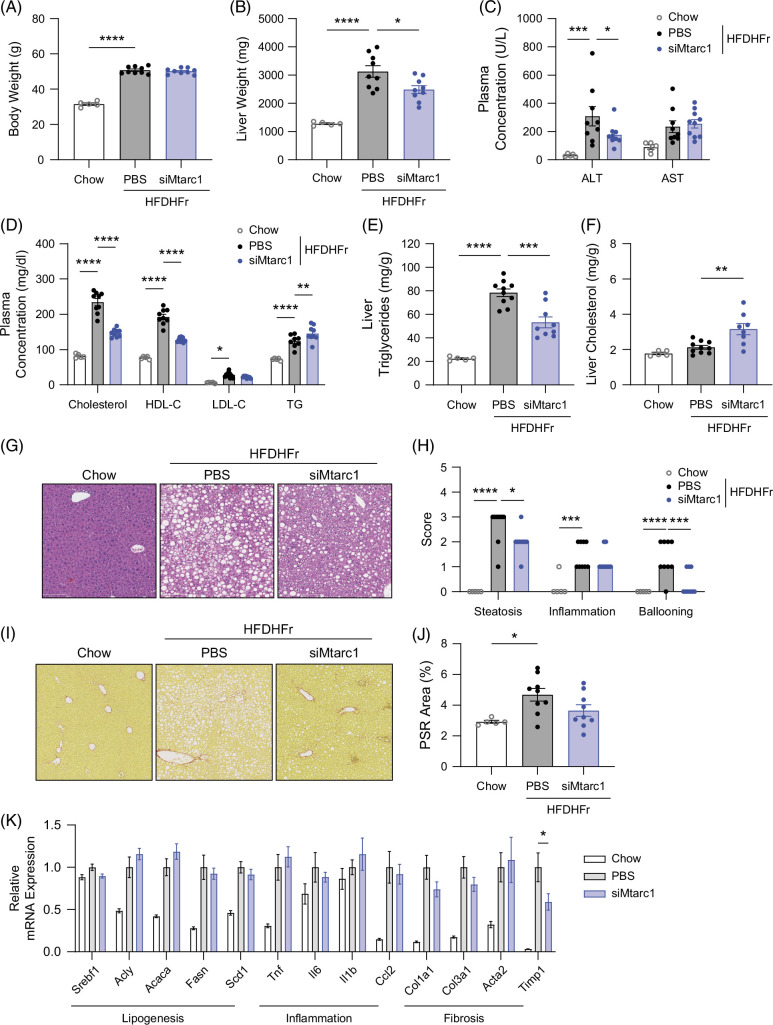
Hepatocyte-specific *Mtarc1* knockdown results in decreased liver fibrosis in male mice fed a high-fat diet supplemented with fructose water. Male C57BL/6J mice were fed an HFD supplemented with 30% w/v fructose water (HFDHFr) for 13 weeks to induce MASH and liver fibrosis then treated with GalNAc-si*Mtarc1* or PBS Q2W for an additional 8 weeks. (A) Body weight and (B) liver weight after 8 weeks of si*Mtarc1* treatment and 21 weeks on diet. (C) Plasma liver enzyme levels and (D) plasma lipid levels at the 20-week time point. (E) Liver triglycerides and (F) liver cholesterol levels. (G) Representative H&E images of liver from PBS and si*Mtarc1* treated mice. (H) The pathologist assessed steatosis, inflammation, and ballooning scores. (I) Representative Picrosirius red (PSR) stained images of liver PBS and si*Mtarc1* treated mice. (J) Quantification of the PSR stained area within the whole liver lobe. (K) Liver mRNA expression of lipogenic, inflammatory, and fibrosis-associated genes. Data are presented as mean +/− SEM. n=10–13 *p<0.05, **p<0.01, ***p<0.001, ****p<0.0001. Abbreviations: HFDHFr, high-fat diet with fructose water; *MTARC1*, mitochondrial amidoxime reducing component 1; TG, triglyceride.

To evaluate whether hepatocyte-specific knockdown of *Mtarc1* protects mice from excess liver fat accumulation, we challenged mice with a highly lipogenic HFDHFr for 12 weeks, followed by biweekly treatment with a GalNAc-conjugated siRNA targeting *Mtarc1* or PBS for 8 weeks. *Mtarc1* was knocked down by over 85% in the liver at both the RNA and protein levels (Supplemental Figure S7, http://links.lww.com/HC9/B878). Compared to normal chow diet-fed mice, mice fed HFDHFr gained significant body weight by the end of the study, but there was no difference in body weight between control and si*Mtarc1-*treated mice (Figure 6A). Although body weight was not affected by si*Mtarc1* treatment, liver weight was significantly reduced (Figure 6B). As expected, the liver function enzyme ALT was elevated in control mice fed HFDHFr, and this was significantly reduced by si*Mtarc1* treatment (Figure 6C). Levels of circulating total and HDL cholesterol were reduced by treatment with si*Mtarc1*, whereas levels of circulating triglycerides were modestly increased (Figure 6D). Conversely, in the liver, triglyceride levels were significantly reduced by si*Mtarc1* treatment (Figure 6E) while cholesterol levels were increased (Figure 6F). The pathologist-evaluated histological assessment showed a significant reduction in both steatosis and ballooning scores after treatment with si*Mtarc1* (Figure 6G, H), and there was a trend toward reduction in liver fibrosis in this model (Figure 6I, J). Profiling a number of genes involved in lipogenesis, inflammation, and fibrosis revealed significant increases in expression in mice fed an HFDHFr compared to normal chow, but the effect of si*Mtarc1* treatment on gene expression changes was limited (Figure 6K). RNA-seq analysis revealed a significant decrease in genes involved in extracellular matrix remodeling and collagen formation (Supplemental Figure S9, http://links.lww.com/HC9/B878). Taken together, these data support that therapeutic intervention with a GalNAc-conjugated siRNA targeting *Mtarc1* in a highly lipogenic diet model of MASH can significantly reduce liver fat accumulation and hepatocyte ballooning.

### Artificial intelligence-powered digital pathology reveals that hepatocyte-specific Mtarc1 knockdown results in decreased macrosteatosis and microsteatosis, zone 2 perisinusoidal fibrosis, and steatosis co-localized fibrosis

**FIGURE 7:**
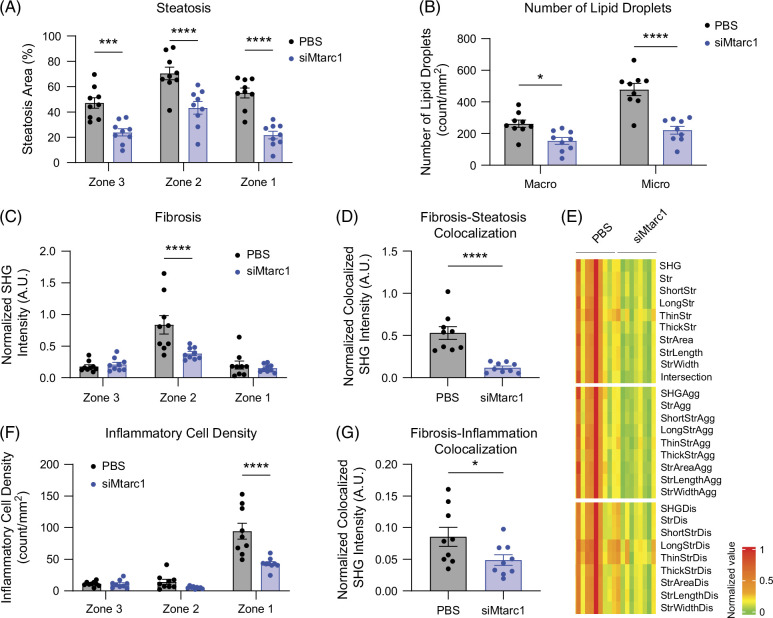
AI-powered digital pathology reveals that hepatocyte-specific *Mtarc1* knockdown results in decreased macrosteatosis and microsteatosis, portal track fibrosis, and steatosis co-localized fibrosis. AI-powered digital pathology was used to analyze histological sections of the liver from male C57BL/6J mice that were fed an HFD supplemented with 30% w/v fructose water (HFDHFr) for 13 weeks to induce MASH and liver fibrosis and then treated with GalNAc-si*Mtarc1* or PBS Q2W for an additional 8 weeks. (A) Area of steatosis within each zonal region of the liver lobule. Zone 3—central vein region, zone 2—perisinusoidal region, and zone 1—portal tract region. (B) Quantification of a number of small micro (<17.5 μm in diameter) and large macro (≥17.5 μm in diameter) lipid droplets per unit area of the liver section. (C) Area of fibrosis within each zonal region of the liver lobule. Zone 3—central vein region, zone 2—perisinusoidal region, zone 1—portal tract region. (D) Quantification of the colocalization of fibrosis with steatosis. (E) Heat map depicting quantitative fibrosis-related parameters and various collagen features from PBS or si*Mtarc1* treated livers in zone 2. (F) Inflammatory cell density within each zonal region of the liver lobule. (G) Quantification of the colocalization of fibrosis with inflammatory cells. Data are presented as mean +/−​​​​​​ SEM. n=10-13 *p<0.05, ***p<0.001, ****p<0.0001. Abbreviations: Agg, aggregated; Dis, distributed; *MTARC1*, mitochondrial amidoxime reducing component 1; SHG, second harmonic generation; Str, string.

Since hepatocyte-specific *Mtarc1* knockdown resulted in significant improvements in histological endpoints evaluated by standard pathology, we next asked whether we could achieve improved granularity in our understanding of these histological endpoints. To this end, we employed artificial intelligence (AI)-powered digital pathology to assess a variety of parameters that cannot be ascertained by traditional pathology including zonation of steatosis and fibrosis within the liver lobule, macrosteatosis and microsteatosis quantitation, collagen fibrillar properties, and co-localization of fibrosis with steatosis or inflammation. We observed that *Mtarc1* knockdown resulted in reduced steatosis in all 3 zones of the liver lobule (Figure 7A) and a decrease in both macrosteatosis and microsteatosis (Figure 7B). In addition, in this HFDHFr model of MASH and fibrosis, fibrosis was predominant in zone 2 (perisinusoidal region) of the liver lobule, and *Mtarc1* knockdown resulted in significantly lower zone 2 fibrosis (Figure 7C) and decreased fibrosis-steatosis co-localization for (Figure 7D). Furthermore, several distinct collagen fibrillar characteristics were reduced when *Mtarc1* is knocked down in the liver including length, thickness, and area (Figure 7E). Finally, we assessed the impact of *Mtarc1* knockdown on inflammatory cell density and observed a significant reduction in inflammatory cells in zone 1 (portal region), where inflammatory cell density was most prominent (Figure 7F), and a significant reduction in fibrosis-inflammation colocalization was observed (Figure 7G). Taken together, our AI-powered digital pathology analysis revealed more detailed insights into the effects of *Mtarc1* knockdown on steatosis, inflammation, and fibrosis, as well as the relative contribution of steatosis and inflammation to fibrosis, which could not be evaluated by traditional histological pathology.

### Hepatocyte-specific Mtarc1 knockdown results in increased circulating triacylglycerol and diacylglycerol species and decreased phosphatidylcholine and phosphatidylinositol lipid species

**FIGURE 8:**
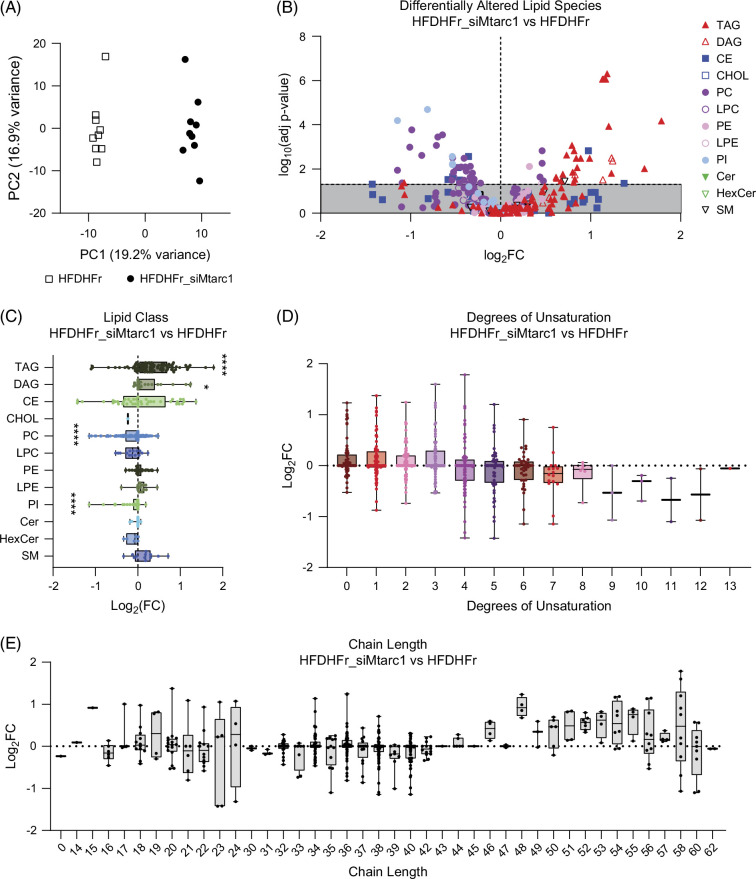
Hepatocyte-specific *Mtarc1* knockdown in male mice fed a high-fat diet supplemented with fructose water alters the circulating lipid profile. Shotgun lipidomics was performed on plasma from male C57BL/6J mice that were fed an HFD supplemented with 30% w/v fructose water (HFDHFr) for 13 weeks to induce MASH and liver fibrosis then treated with GalNAc-si*Mtarc1* or PBS Q2W for an additional 8 weeks. (A) Principal component analysis comparing control (HFDHFr) with si*Mtarc1-*treated mice (HFDHFr_si*Mtarc1*). (B) Volcano plot of differentially altered lipid species categorized by lipid class. (C) Fold changes of lipid species characterized by class in si*Mtarc1-*treated samples compared with control. (D) Degrees of unsaturation of all lipid species. (E) Carbon chain length of all lipid species. n=9 samples per treatment group. Adjusted *p<0.05, **** *p*<0.0001. Abbreviations: CE, cholesterol ester; Cer, ceramide; CHOL, cholesterol; DAG, diacylglycerol; HexCer, hexosylceramide; HFDHFr, high-fat diet with fructose water; LPC, lysophosphosphatidylcholine; LPE, lysophosphatidylethanolamine; *MTARC1*, mitochondrial amidoxime reducing component 1; PC, phosphatidylcholine; PE, phophatidylethanolamine; PI, phosphatidylinositol; SM, sphingomyelin; TAG, triacylglycerol.

To identify more systemic changes in response to the loss of *Mtarc1* in the liver, we assessed an immune monitoring panel in plasma samples from mice fed a high-fat diet supplemented with fructose water to determine whether there were significant changes in up to 48 different cytokines, chemokine, or growth factor targets; however, no differences were detected between the treatment groups for all analytes tested (Supplemental Figure S10, http://links.lww.com/HC9/B878). We also performed untargeted lipidomics of plasma samples from the same mice. We observed a distinct separation between the control and si*Mtarc1-*treated groups by principal component analysis (Figure 8A) and identified several lipid species that were differentially changed between treatment groups (Figure 8B). Of the 515 lipids quantified, 74 were differentially changed on si*Mtarc1* treatment, including 35 that were more abundant and 39 that were less abundant. Several lipid classes were significantly changed on si*Mtarc1* treatment, including triacylglycerol and diacylglycerol lipid species that were found to be more abundant, and phosphatidylcholine (PC) and phosphatidylinositol lipid species that were found to be less abundant (Figure 8C). We also assessed other characteristics of the lipids, including degree of unsaturation and carbon chain length, and found no significant differences; however, we observed a trend towards increased chain length in samples treated with si*Mtarc1* (Figure 8D, E). Taken together, evaluating the changes in circulating biomarkers revealed no differences in immune markers but a significantly altered plasma lipidome in response to hepatocyte-specific knockdown of *Mtarc1*.

## DISCUSSION

Several recent studies have characterized the role of mARC1 in mouse and cell culture models. The current study is the first to demonstrate that loss of mARC1 in vivo, by both genetic KO or siRNA-mediated knockdown, results in protection from disease progression in 4 distinct models of MASH and liver fibrosis and that the timing of intervention is critical to observe a protective phenotype. These results are consistent with human genetics where carriers of *MTARC1* loss of function variants are protected from all-cause cirrhosis^12^ but contrasts with recent findings by Smagris et al^31^ who observed no effect of the genetic deletion of *Mtarc1* on steatosis and fibrosis endpoints in several models of MASH in mice. These different results may be due to different methods for KO mouse generation where our model completely removes all selection cassettes after Cre recombination while their model retains a β-galactosidase gene cassette in the flanking intron^31^ but are more likely due to differences in the models and time points used to assess the impact of loss of mARC1 on the liver. For example, in our study, we demonstrated a significant impact of *Mtarc1* KO on liver fibrosis in models with moderate to severe disease, while Smagris et al^31^ assessed the impact of *Mtarc1* KO on liver phenotypes at later time points when the disease burden may have been too severe for loss of mARC1 to overcome. The temporal effect of loss of mARC1 is most evident in our siRNA therapeutic intervention model where intervention with knockdown of mARC1 at week 16 improved liver fibrosis but intervention at week 24 did not (Figure 6 and Supplemental Figure S6, http://links.lww.com/HC9/B878). A recent perspective piece highlighted the importance of fit-for-purpose model selection and temporal profiling of interventions in well-established MASH and liver fibrosis models^36^ and the differences between the 2 independent studies characterizing the effect of *Mtarc1* KO emphasize the importance of this. Importantly, coupling the results from our *Mtarc1* KO model and our siRNA therapeutic intervention model has revealed that loss of mARC1 is hepatoprotective in both preventative and therapeutic paradigms helping to refine our understanding of mARC1 biology.

In cell culture models, we have shown that the hepatoprotective p.A165T mARC1 variant is decreased at the protein level when overexpressed, which is consistent with several previous reports expressing this variant in a variety of cell lines including HEK293, Huh7, HepG2, and U-138MG^29^,^31^,^33^,^34^,^35^ and recent studies have reported that this decrease in p.A165T protein abundance is due to increased ubiquitination and proteasomal degradation of this variant and reduced protein localization to mitochondria.^33^,^34^,^35^ However, in this study we did not observe a difference in mitochondrial localization of the mARC1 A165T variant, which is consistent with another study demonstrating similar endomembrane localization using a biochemical fractionation technique.^33^ Interestingly, in the current study, we observed a significant discrepancy between *Mtarc1* mRNA and mARC1 protein levels when p.A165T mARC1 is overexpressed in cells or mouse liver, consistent with the notion that there may be post-transcriptional and/or post-translational regulation of this variant. Another study revealed similar discrepancies between *Mtarc1* mRNA and mARC1 protein levels in mouse liver during fasting and high-fat diet feeding studies.^37^ Further studies to explore the regulation of mARC1 in the liver at the transcriptional, post-transcriptional, and post-translational levels are warranted to better understand the biology of mARC1. The differences in results from cell culture models described to date testing mARC1 variant expression levels and localization can likely be attributed to different cell lines used, different approaches to express mARC1 variants, and different primary antibodies for detection of mARC1 by immunoblot and highlights the practical limitations of studying mARC1 protein function using cell culture models. In human liver samples, the effect of p.A165T on mARC1 protein levels is more ambiguous with one study demonstrating no difference in protein abundance between 165A and 165T variants in pediatric livers by mass spectrometry,^32^ while another study demonstrated a ~50% decrease in 165T protein levels in human liver samples by immunoblot^31^ similar to what has been described in cell culture models. More studies using human liver biopsies are needed to fully understand the impact of the p.A165T variant on mARC1 protein levels in the human liver.

As highlighted above, understanding the molecular mechanisms of action of mARC1 in the liver has proven challenging. To date, the endogenous substrate(s) of mARC1 enzymatic activity have not yet been identified in physiological systems. In this study, we demonstrated that knockdown of mARC1 protected cells from lipotoxic stress induced by fatty acids, such as palmitate (Figure 2). These results are supported by independent findings demonstrating that loss of mARC1 can reduce oxidative stress and improve the GSH:GSSG ratio in primary human hepatocyte spheroids and overexpression of the mARC1 protective allele improves mitochondrial membrane potential compared to overexpression of the risk allele.^29^ We also demonstrated a similar impact of mARC2 loss on hepatoprotective effects, underscoring a comparable function between mARC1 and mARC2 in the liver. A recent report suggests that it is mARC2, not mARC1, retains most of the N-reductive capacity in the mouse liver^31^ although these authors only assessed the activity on a single, nonphysiological substrate. It remains unknown whether mARC1 can catalyze the reduction of an unidentified substrate in the mouse liver. It is likely that understanding the distinct substrate preferences of these 2 close paralogs will allow for a better understanding of their biology. In the present study, one clear difference between the loss of mARC1 and mARC2 was their impact on cellular bioenergetics. Increased oxygen consumption and glycolytic rate were only observed in cells where mARC1, but not mARC2, had been knocked down, but how the loss of mARC1 improves cellular bioenergetics is still unclear. Further studies are necessary to elucidate how the molecular mechanisms of mARC1 and mARC2 differ, and this will be critical for understanding how they differentially contribute to liver pathogenesis.

Since disease progression to fibrosis is associated with an increased risk of mortality,^9^,^10^ regression of fibrosis is thought to decrease the risk of disease progression, and loss of mARC1 in our preclinical models significantly reduced fibrosis, we sought to characterize the histopathological features of fibrosis more thoroughly using AI-powered digital pathology. Clinically, regression of fibrosis, specifically in zone 2 (perisinusoidal region), may have important implications for patient outcomes as demonstrated by the AI-powered digital pathology analysis of the FLIGHT-FXR study.^38^,^39^,^40^ The subjects in this study who responded to tropifexor treatment showed the greatest regression of fibrosis in zone 2. In addition, subjects who showed improvements in steatosis and ballooning also showed improvements in fibrosis within the same co-localized region. Remarkably, hepatocyte-specific knockdown of mARC1 in mice resulted in a similar effect on liver fibrosis as observed in the FLIGHT-FXR study with significantly decreased zone 2 fibrosis and reduced co-localization steatosis and inflammation with fibrosis revealing the translational potential of targeting mARC1 for the treatment of liver fibrosis. Additional analysis of clinical programs, such as MAESTRO-NASH, using AI-digital pathology will help further refine our understanding of the histological features of fibrosis regression and help define the key features of fibrosis to be targeted in the clinic.

Untargeted plasma lipidomics after hepatocyte-specific mARC1 knockdown revealed a significant alteration in the plasma lipidome, including increased triacylglycerol and diacylglycerol species and decreased PC and phosphatidylinositol lipids (Figure 8). Similar lipidomics data from large cohorts of human carriers of several MASH-associated SNPs revealed changes in PC and diacylglycerol species in mARC1 p.A165T carriers following the same directionality observed in our study and consistent with A165T conferring loss of function.^41^ Interestingly, another study in humans profiled the liver lipidome of carriers of the mARC1 p.A165Tvariant and found an enrichment of PC species in the liver.^42^ Together, these studies revealed an inverse relationship between liver and circulating PC species in p.A165T carriers. Importantly, patients with MASLD and MASH have a lower PC to PE ratio than healthy subjects,^43^ and rodent studies support that a lower hepatic ratio of PC to PE contributes to the development of hepatic steatosis.^44^ Hepatic PCs were also significantly lower in homozygous carriers of *PNPLA3* I148M and enriched in carriers of *HSD17B13* TA variant, consistent with their deleterious and protective effects on MASLD progression, respectively.^45^,^46^ Taken together, our preclinical data and the data from human p.A165T carriers suggest that loss of mARC1 may favor increased levels of PC in the liver, resulting in decreased PC levels in the plasma, improving the PC to PE ratio in the liver, and preventing disease progression.

In summary, we have demonstrated that the loss of mARC1 is hepatoprotective and can protect cells from lipotoxic stress and improve cellular bioenergetics. Indeed, in mouse models of MASH and liver fibrosis, loss of mARC1 can reduce liver steatosis, inflammation, and fibrosis and has a profound effect on the plasma lipidome. Together, these results build on the previously defined role of mARC1 as an important N-reductive enzyme in the liver and support the hypothesis that therapeutic targeting of mARC1 is an innovative strategy for the treatment of late-stage liver disease. Further mechanistic studies are warranted to characterize the molecular mechanism of mARC1 action in the liver to refine our understanding of this target in the pathogenesis of MASLD and to explore whether the loss of mARC1 may have additive or synergistic effects with other liver-directed therapies.

## Supplementary Material
