# Supplementary material for: Loss of mitochondrial amidoxime-reducing component 1 (mARC1) prevents disease progression by reducing fibrosis in multiple mouse models of chronic liver disease
Source: Hepatol Commun. 2025 Feb 10;9(2):e0637. doi: 10.1097/HC9.0000000000000637 (PMC11809980; doi:10.1097/HC9.0000000000000637)
Supplement: Supplementary file 1 [file hc9-9-e0637-s001.docx]

**Supplemental Information**

**Materials and Methods**

**ANIMAL STUDIES**

**Human mARC1 adeno-associated virus**

The coding region of human *MTARC1* (NM_022746.4) and mutants A165T, M187K, R200Ter, C273A, together with EagI and KOZAK sequence GCCACC at 5’ end and HindIII at 3’end, were synthesized and cloned into pUC57 shuttle plasmid (Azenta Life Sciences). R200Ter is a truncated form that encodes the first 200 amino acids of human mARC1. The synthesized human *MTARC1*, A165T, M187K, R200Ter, and C273A were subsequently subcloned into EagI and HindIII of a pAAV cis plasmid that contains the ubiquitous CB promoter. The self-complementary recombinant AAV8 vectors were generated with the helper-free triple plasmid transfection method in HEK293 cells and purified using double cesium chloride gradient centrifugation by the Viral Vector Core of University of Massachusetts Medical School. The titers were determined via real-time PCR analysis. Mice were administered 3x10^11^ GC of indicated AAV via tail vein injection and liver expression was evaluated 11 days later.

**Diet and Liver Injury Models**

Several MASH-inducing models have been used including the Gubra Amylin NASH diet (GAN), high-fat diet supplemented with fructose water (HFDHFr), and choline-deficient amino acid-defined diet (CDAA-HFD). For GAN diet studies, male mice at 8 weeks of age were randomized into groups with equivalent body weights and were fed a high-fat, high-fructose, high-cholesterol GAN diet (Research Diets D09100310) for up to 32 weeks. For GAN Diet siRNA studies, after 16 or 24 weeks on diet, mice were treated with 10 mg/kg mARC1 targeting siRNA^1^ or PBS control Q2W for 8 weeks. For HFDHFr diet studies, mice were fed a high fat diet (Research Diets D12492) supplemented with fructose water (30% w/v fructose) for up to 20 weeks. For siRNA studies, after 12 weeks on diet, mice were treated with 10 mg/kg mARC1 targeting siRNA or PBS control Q2W for 8 weeks. For CDAA-HFD studies, mice were fed a CDAA-HFD (Research Diets A06071302) for 12 weeks. At the end of a study, mice were anesthetized using isoflurane prior to terminal exsanguination followed by bilateral thoracotomy. For CCl_4_ studies, mice were dosed intraperitoneally with CCl_4_ diluted in olive oil at a dose of 0.1 or 0.5 ul/g twice weekly for 4 weeks. Blood was collected 24 hours after dose number 4 to assess the acute hepatotoxic effect of CCl_4_ on liver enzymes. After 4 weeks, livers were collected, and liver fibrosis was assessed by histology.

**Plasma and tissue analysis**

Blood was collected from animals at the indicated timepoints by cardiac puncture and transferred into K2-EDTA tubes following a 5 hour fast. ALT, AST, ALP, total cholesterol, LDL-C, HDL-C, and triglycerides were measured using a Cobas c 311 Clinical Analyzer (Roche). Plasma cytokines and chemokines were analyzed using the Immune Monitoring 48-Plex Mouse ProcartaPlex™ Panel (Thermo Fisher EPX480-20834-901) per the manufacturer’s protocol. For liver lipid analysis, ~40 mg of tissue was homogenized in 5% IGEPAL CA-630, heated to 80-100°C twice, centrifuged, and the extracted lipids from the supernatant were measured using the Cobas c 311 Clinical Analyzer triglyceride and total cholesterol kits.

**Histology and Pathology**

The left lateral lobe of the liver was fixed, embedded in paraffin, sectioned, and stained with hematoxylin and eosin (H&E) and picrosirius red (PSR). All stained slides were examined by light microscopy and digitally scanned at 20X brightfield. A trained pathologist used the MASH Clinical Research Network (CRN) scoring system to obtain the MASLD activity score. The PSR stained area was quantified by using whole-slide image analysis. The observer was blinded to the samples.

**AI-powered Digital Pathology**

Fibrosis and steatosis detection: Unstained formalin-fixed paraffin-embedded (FFPE) tissue slides (section thickness: 5 um) were scanned using the Genesis® 200 system (HistoIndex), which uses an ultrafast femtosecond laser, emitting photons to excite the unstained tissue sample at 780 nm. Second Harmonic Generation (SHG) signals at 390 nm and Two-Photon Excited Fluorescence (TPEF) signals at 550 nm were then collected at two photomultiplier tubes (PMT). The monochromic signals were colorized and subsequently merged to form SHG/TPEF image, giving a resolution of 0.39 um/pixel. TPEF signals (red in color) provide visualization of background liver architecture while SHG signals (green color) identify collagen fibers. AI-based image analysis was performed to identify liver zone-specific 100 fibrosis features and 45 steatosis features^2^, as well as the colocalization between fibrosis and steatosis^3^.

Inflammatory cell detection: Following the SHG/TPEF scanning, the same slides were stained using Hematoxylin & Eosin (H&E). The whole slide images (WSI) were acquired using Vectra Polaris at 40x. Inflammatory cells were detected and quantified on the H&E images using a two-stage AI pipeline that combines supervised and unsupervised approaches, based on spatial context representation^4^. In the first stage, the H&E images were divided into patches, and evaluated with a pretrained deep learning pipeline to obtain the centroids and categories of the cells. It integrates several independent modules, including feature extractor, cell detection, cell classification, spatial context categorization, and deep clustering. In the second stage, segmentation of cells with StarDist^5,6^ was performed and matched with the cell detection and classification results from the first stage. Inflammatory cell density (i.e., the number of inflammatory cells per unit area) was then calculated. To assess the colocalization between inflammatory cells and fibrosis, the H&E images were registered to the corresponding SHG/TPEF images. The amount of fibrosis in the vicinity of inflammatory cells was quantified.

Liver zone-specific quantification: Extracted parameters from H&E images were also quantified in separate liver zone. For liver zone (Zone 3/CV, Zone 2/PS, Zone 1/PT) detection, the classification was performed in two stages: liver vein segmentation, followed by classification of each detected vein into portal vein or central vein. In the first stage we divided the tissue area of each H&E-stained WSIs into patches of size 512 x 512 with 50% overlap at 10x resolution and normalized them using Macenko method^7^. We then deployed a pretrained U-Net model^8^ with ResNet-50 ^9^ backbone to segment all veins found in these patches and combined overlapping veins together. In the second stage, we extracted the region within the bounding box of each vein dilated by 100 um and deployed a pretrained Attention-based Deep Multiple Instance Learning^10^ approach with ResNet-18 backbone to classify each vein.

**Illumina Stranded Total RNA Prep and Novaseq Sequencing**

RNA from liver was prepared using QIAzol reagent followed by chloroform extraction (Qiagen #79306). RNA was purified using the RNeasy mini kit with a DNAse digestion (Qiagen #74416). For each sample, total RNA was normalized to 100 ng before undergoing library preparation using the Illumina Stranded Total RNA Prep Ligation with Ribo-Zero Plus kit (Illumina) per manufacturer’s instructions. The Agilent Bioanalyzer (Agilent Technologies) and Qubit Fluorometer (Thermo Fisher Scientific) were used to qualify and quantify libraries prior to pooling samples. Samples were sequenced on the Illumina Novaseq 6000 using 2x100 cycles, per manufacturer’s instructions (Illumina).

**RNA sequencing analysis**

Following DNA sequencing and read mapping to the mouse GRCm39 genome, only genes mapping to coding regions were retained, the number of read counts per gene was calculated, and the resulting counts were compiled into a read count table. This table was then filtered row-wise to retain only genes with an FPKM value greater than or equal to one in at least half of the samples. Using these tables, DEseq2 analysis was performed to calculate fold change values. Genes with an adjusted p-value < 0.05 were considered significant^11^. These results were visualized using ggplot2 and ComplexHeatmap packages^12^. Pathway overrepresentation analyses, such as those illustrated in figure Supp Figure 8E, were performed using the clusterProfiler package, while gene set enrichment analyses (GSEA), such as those illustrated in Supp Figure 8D, were performed using the fgsea package^13,14^. PCA was performed using several steps. First, a log transformation was performed on the filtered read counts. The resulting table was ordered in decreasing order by the row-variance across all samples then PCA analysis was performed on the top 500 genes using the stats package. Data has been deposited in the Gene Expression Omnibus repository (GSE272274).

**Plasma Lipidomics Analysis**

1 µL of plasma was analyzed using Shotgun Lipidomics platform by Lipotype GmbH (Dresden, Germany), as described previously^15^. Lipidomics analyses were performed in R using the lipidr package^16^. After importing the data into this package, the data was normalized using Probabilistic Quotient Normalization (PQN) prior to performing additional analyses. PCA analyses were performed using Orthogonal Partial Least Square Discriminant Analysis (OPLS-DA) along with the default settings as described in the lipidr documentation. Differential lipid abundance analysis was performed using limma^17^.

**CELL STUDIES**

**Expression Constructs**

Expression constructs for mARC1 variants were generated from synthetic oligonucleotides. The fragment was inserted into pCDNA3.1-TOPO or pcDNA6.2 c-EmGFP/YFP-DEST for GFP tagged constructs. The plasmid DNA was purified from transformed bacteria and the concentration determined by UV spectroscopy. The final construct was verified by sequencing. The sequence identity within the insertion sites was 100%.

**siRNA**

siRNA targeting human for mARC1, mARC2, and negative control were purchased from ThermoFisher (mARC1 #s34874, mARC2 #s29949, NC siRNA #4390844).

**Cell Culture**

HuH-7 (Huh7) cells were purchased from JCRB (JCRB040) and were maintained in DMEM low glucose supplemented with 10% heat inactivated FBS (Gibco A56708) at 37°C in 5% CO_2_. HEK293 cells were purchased from ATCC (CRL-1573) and were maintained in DMEM supplemented with 10% FBS at 37°C in 5% CO_2_. For mARC1 variant expression studies, an equal amount of plasmid DNA was transfected into HEK293 or Huh7 cells using Lipofectamine 3000 (ThermoFisher L3000001) per manufacturer’s protocol and cells were harvested 48 hours later for immunoblot or RNA analysis.

For cell-based lipotoxic stress studies, 5,000 Huh7 cells per well were plated in DMEM low glucose supplemented with 10% FBS in a 96 well tissue culture plate. After 24 hours, cells were transfected with siRNA (described above) using Lipofectamine RNAiMAX (ThermoFisher #13778075). After 24 hours, cells were treated with 400 uM of BSA-oleate and/or BSA-palmitate (Cayman Chemical #29557, #29558) for 24 hours. Cells were collected for qPCR analysis using the cells-to-Ct kit (ThermoFisher #AM1729), mitochondrial superoxide production using the MitoSox Red kit (ThermoFisher #M36007) or membrane potential using the mitoprobe JC-1 assay kit (ThermoFisher #M34152) as per manufacturer’s protocol.

**Fluorescence Microscopy**

Huh7 cells were plated in 96 well plates and the following day, cells were transfected with mARC1-GFP plasmids using Lipofectamine 3000 (ThermoFisher L3000001) according to manufacturer’s protocol. 24 hours later, cells were stained with MitoTracker Red CMXRos (ThermoFisher M7512) for 30 minutes before nuclei were stained using Hoechst 33342 (ThermoFisher H1399) for 5 minutes. Live cell imaging was performed using Opera Phenix High-Content Screening System with 63x objective.

**Seahorse Analysis**

Seahorse assays were run on the Seahorse XF Pro Metabolic Analyzer. Huh7 cells were assayed using the Seahorse XF Cell Mito Stress Test Kit (Agilent #103015-100). Huh7 cells were plated at 13,000 cells per well in a Seahorse XF Pro M Cell Culture Microplate (Agilent #103774-100) and 24 hours later, cells were transfected with siRNA as described above. After 48 hours, the Seahorse Cell Mito Stress Test assay was run according to manufacturer’s protocol with 1.5 μM oligomycin, 0.5 μM FCCP, and 0.5 μM rotenone/antimycin A. Assays were normalized to DNA content using CyQuant (ThermoFisher #C7026) after the assay was complete.

**Immunoblot Analysis**

*Liver homogenates*

Liver homogenates were prepared in ice cold RIPA buffer with protease and phosphatase inhibitors (ThermoFisher # 78442). Total protein was quantified using Pierce BCA Protein assay kit (ThermoFisher #23225). 20 ug of whole liver homogenate was loaded onto a NuPAGE 4-12% BisTris gel (ThermoFisher #WG1402BOX) and gel electrophoresis was performed. Gels were transferred to nitrocellulose membranes using the iBlot2 dry transfer system (ThermoFisher # IB23001) and membranes were blocked using LICOR TBS blocking buffer (LICOR #927-66003). After blocking, membranes were incubated with primary antibodies listed in Supplemental Table 1 overnight. Membranes were washed and then incubated for 1 hour at room temperature with secondary antibodies IRDye 680RD Goat anti-Mouse IgG (Licor #926-68070) and IRDye 800CW Goat anti-Rabbit IgG (Licor #926-32213). Membranes were visualized on the Licor Odyssey CLx. Blots were quantified using densitometry analysis in Image Studio (Licor).

*Cell lysates*

Cell lysates were prepared in ice cold RIPA buffer with protease and phosphatase inhibitors (ThermoFisher # 78442). Total protein was quantified using Pierce BCA Protein assay kit (ThermoFisher #23225). 10 ug of cell lysate was loaded onto a NuPAGE 4-12% BisTris gel (ThermoFisher #WG1402BOX) and gel electrophoresis was performed. Gels were transferred to nitrocellulose membranes using the iBlot2 dry transfer system (ThermoFisher # IB23001) and membranes were blocked using LICOR TBS blocking buffer (LICOR #927-66003). After blocking, membranes were incubated with primary antibodies listed in Supplemental Table 1 overnight. Membranes were washed and then incubated for 1 hour at room temperature with secondary antibodies IRDye 680RD Goat anti-Mouse IgG (Licor #926-68070) and IRDye 800CW Goat anti-Rabbit IgG (Licor #926-32213). Membranes were visualized on the Licor Odyssey CLx. Blots were quantified using densitometry analysis in Image Studio (Licor).

**qPCR Analysis**

*Liver*

RNA from liver was prepared using QIAzol reagent followed by chloroform extraction (Qiagen #79306). RNA was purified using the RNeasy mini kit with a DNAse digestion (Qiagen #74416). 1 μg of RNA was converted to cDNA using SuperScript VILO cDNA Synthesis Kit (Thermo Fisher #11754050) and qPCR was performed using TaqMan Fast Advanced Master Mix (ThermoFisher #4444557) on a Viia7 instrument (Applied Biosystems). Taqman probes (ThermoFisher) used to assess gene expression levels are listed in Supplemental Table 2 and Tbp was used as a housekeeping gene.

*Cells*

Cells were washed with ice cold PBS. RNA from cells was extracted using the RNeasy mini kit with a DNAse digestion (Qiagen #74416). 1 μg of RNA was converted to cDNA using SuperScript VILO cDNA Synthesis Kit (Thermo Fisher #11754050) and qPCR was performed using TaqMan Fast Advanced Master Mix (ThermoFisher #4444557) on a Viia7 instrument (Applied Biosystems). Taqman probes (ThermoFisher) used to assess gene expression levels are listed in Supplemental Table 2 and Tbp was used as a housekeeping gene.

**Supplemental Results**

***Generation of Mtarc1 knockout mice***

To further explore whether the loss of mARC1 confers protective effects in the liver, we generated *Mtarc1* knockout mice. We generated a constitutive global knockout of *Mtarc1*, also known as *Mosc1*, by homologous recombination in embryonic stem cells (hereafter referred to as *Mtarc1* KO) (Supp. Figure 3A). We profiled the mRNA expression of *Mtarc1* and *Mtarc2* in various tissues including the liver, inguinal adipose tissue, gonadal adipose tissue, brown adipose tissue, gastrocnemius muscle, heart, lung, kidney, spleen, and brain. *Mtarc1* was most abundantly expressed in mouse liver and *Mtarc1* mRNA was not detected in any of the profiled knockout tissues (Supplementary Figure 3B). In addition, there was no compensatory upregulation of *Mtarc2* mRNA in response to loss of *Mtarc1* in any of the tissues tested (Supp. Figure 3C). Consistent with the gene expression patterns, mARC1 protein was not detected in any of the *Mtarc1* KO tissues by immunoblotting and was most abundant in the liver in WT mice (Supp Figure 3D).

***Mtarc1 gene deletion results in decreased liver fibrosis in CDAA-HFD and chronic CCl_4_ liver injury models***

To further evaluate the effect of *Mtarc1* gene deletion on liver fibrosis, WT and *Mtarc1* KO mice were challenged with a choline-deficient, amino acid-defined high fat diet (CDAA-HFD) for 12 weeks to induce significant fibrosis. At the end of the study, no differences in body weight were observed, but liver weight was significantly decreased in *Mtarc1* KO mice (Supplementary Figure 4A-B), and there were no differences observed in liver enzymes or plasma lipids between genotypes (Supplementary Figure 4C-F). Based on histological analysis, there was no effect of *Mtarc1* KO on MAS, steatosis, inflammation, or ballooning scores (Supplementary Figure 4G-I), but a significant decrease in PSR area and liver fibrosis (Supplementary Figure 4J-K). We confirmed by qPCR that there was no expression of *Mtarc1* mRNA and no compensatory upregulation of *Mtarc2* mRNA in *Mtarc1* KO livers in this model (Supplementary Figure 4L). In addition, we observed no significant differences in lipogenic, inflammatory, or fibrotic gene expression. Taken together, we have demonstrated in a more severe model of MASH that the loss of mARC1 can significantly reduce liver fibrosis.

To determine whether the effect of *Mtarc1* gene deletion on liver fibrosis could be observed independently of metabolic injury driven by high-fat feeding, we challenged mice with chronic exposure to hepatotoxin CCl_4_ at low and high doses of 0.1 or 0.5 ul/g. After 4 weeks of chronic treatment, we observed no differences in body or liver weight in *Mtarc1* KO mice compared to WT mice (Supplementary Figure 5A-B) and *Mtarc1* was not expressed in KO liver (Supplementary Figure 5C). Knockout of mARC1 resulted in a significant decrease in ALT, but not AST at the 0.1 ul/g dose of CCl_4_ and no differences were observed between genotypes at the 0.5 ul/g dose (Supplementary Figure 5D-E). Finally, we assessed the impact of loss of mARC1 on liver fibrosis in response to CCl_4_ treatment and observed no impact on *Col3a1* gene expression (Supplementary Figure 5F), but significantly decreased fibrosis measured by PSR-stained area in the 0.1 ul/g treated group (Supplementary Figure 5G-H). Taken together, these data demonstrate that the loss of mARC1 can protect the liver from CDAA-HFD or CCl_4_-induced liver fibrosis.

***Hepatocyte-specific Mtarc1 knockdown does not reduce fibrosis in after 32 weeks on the GAN Diet***

To understand the impact of disease burden on mARC1 efficacy, we tested whether knocking down *Mtarc1* at a later timepoint with a higher disease burden would have a similar impact. In this paradigm, mice were fed the GAN diet for 24 weeks, followed by a biweekly treatment with siRNA for an additional 8 weeks. Similar to the knockdown of *Mtarc1* starting at 16 weeks, we observed robust knockdown of *Mtarc1* (Supplementary Figure 6A), no changes in liver enzymes (Supp Figure 6B), decreased plasma cholesterol levels (Supplementary Figure 6C), no changes in body weight (Supp Figure 6D), decreased liver weight (Supplementary Figure 6E), and no changes in the MASLD activity score, steatosis, inflammation, or ballooning (Supplementary Figure 6F-H). However, contrary to knocking down *Mtarc1* at an earlier timepoint, we did not observe a significant decrease in liver fibrosis (Supplementary Figure 6I-J) suggesting that the timing of therapeutic intervention is important for its impact on liver fibrosis endpoints in this model.

***Hepatocyte-specific Mtarc1 knockdown does not reduce fibrosis in the CDAA-HFD model***

To further evaluate the effect of *Mtarc1* hepatocyte-specific knockdown on liver fibrosis, mice were challenged mice with a choline-deficient, amino acid-defined high fat diet (CDAA-HFD) for 4 weeks to induce significant steatosis, inflammation, and the onset of fibrosis. After 4 weeks on diet, mice were treated with *Mtarc1* siRNA for an additional 4 weeks. At the end of the study, *Mtarc1* mRNA levels were significantly decreased in the livers of control treated mice on the CDAA-HFD, and *Mtarc1* levels were further decreased by 50% in the *Mtarc1* siRNA treated mice (Supplementary Figure 8A). No differences in body weight were observed, and liver weight was unchanged between control and *Mtarc1* siRNA treated mice (Supplementary Figure 8B-C). In addition, there were no differences observed in the levels of liver enzyme ALT between control or Mtarc1 siRNA treated mice (Supplementary Figure 8D). Based on histological analysis, there was no effect of *Mtarc1* KO on MASLD activity score (Supplementary Figure 8E) or liver fibrosis (Supplementary Figure 8F). Taken together, we have demonstrated that *Mtarc1* hepatocyte specific siRNA is less effective at silencing *Mtarc1* in the CDAA-HFD model and there was no effect of Mtarc1 knockdown on liver endpoints such as MASLD activity score and liver fibrosis.

***Liver RNA-seq reveals that hepatocyte-specific Mtarc1 knockdown results in alterations in extracellular matrix organization and collagen formation pathways***

To further refine our understanding of mARC1 function in the liver, we performed RNA-seq on the livers of mice fed a high-fat diet supplemented with fructose water for 12 weeks, followed by treatment GalNAc-conjugated siRNA targeting *Mtarc1* or control for 8 weeks. A total of 7041 genes were differentially regulated in response to HFDHFr compared with the normal chow diet, with nearly equal numbers being upregulated (3537) and downregulated (3504) (Supplementary Figure 9A-B). In response to loss of *Mtarc1*, 4056 genes were differentially regulated and a total of 1595 genes were differentially regulated in the opposite direction of diet-induced gene expression changes (Supplementary Figure 8A-B) suggesting a reversal of the pathogenic pathways regulated by the MASH-inducing diet. Principal component analysis (PCA) revealed a distinct separation between the HFDHFr control and HFDHFr plus si*Mtarc1* treatment groups (Supplementary Figure 9C). Gene set enrichment analysis revealed a significant down regulation in the pathways involved in extracellular matrix (ECM) organization and collagen formation in response to *Mtarc1* knockdown in the liver consistent with the effects of si*Mtarc1* on reducing liver fibrosis (Supp Figure 9D). Interestingly, several pathways involved in eukaryotic and viral translation were upregulated in response to si*Mtarc1* treatment (Supplementary Figure 9D). Pathway analysis revealed distinct clustering of chow, HFDHFr control, and HFDHFr with si*Mtarc1* treatment groups with several pathways involved in cellular responses to stress altered in the si*Mtarc1* treated livers (Supplementary Figure 9E).

**
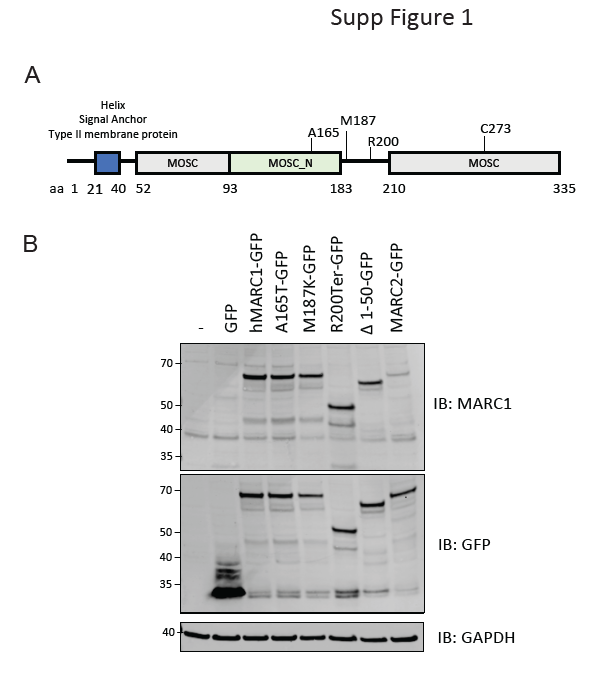
**

**Supplemental Figure 1: Human mARC1 C-terminal GFP constructs were expressed in Huh7 cells.** (A) Schematic of mARC1 protein domains and location of genetic variant point mutations. (B) Immunoblot of mARC1, GFP, and GAPDH.

**
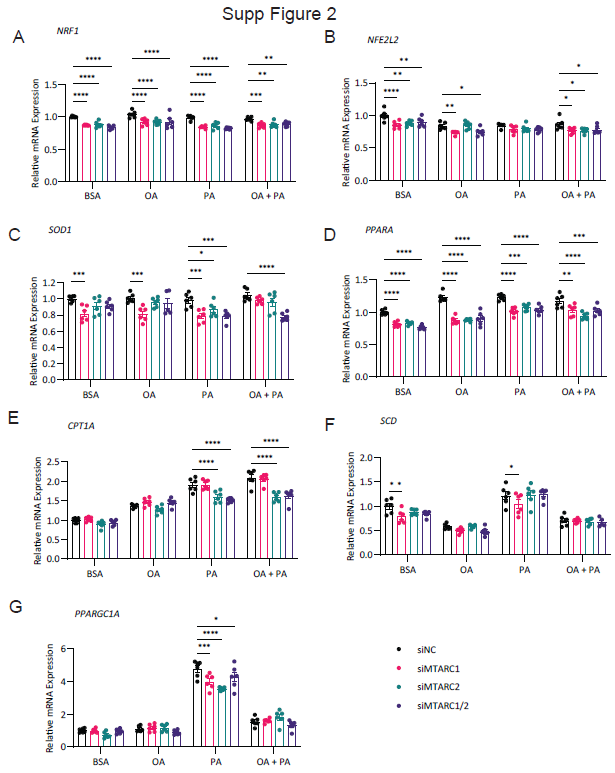
**

**Supplemental Figure 2: Gene expression changes in Huh7 cells after knockdown of *MTARC1* and/or *MTARC2* and treatment with BSA, OA, PA, OA and PA for 24 hours**. Expression of genes involved in oxidative stress (A) *NRF1*, (B) *NFE2L2*, (C) *SOD1*, lipid metabolism (D) *PPARA*, (E) *CPT1A*, (F) *SCD*, and mitochondrial biogenesis (G) *PPARGC1A*.

**
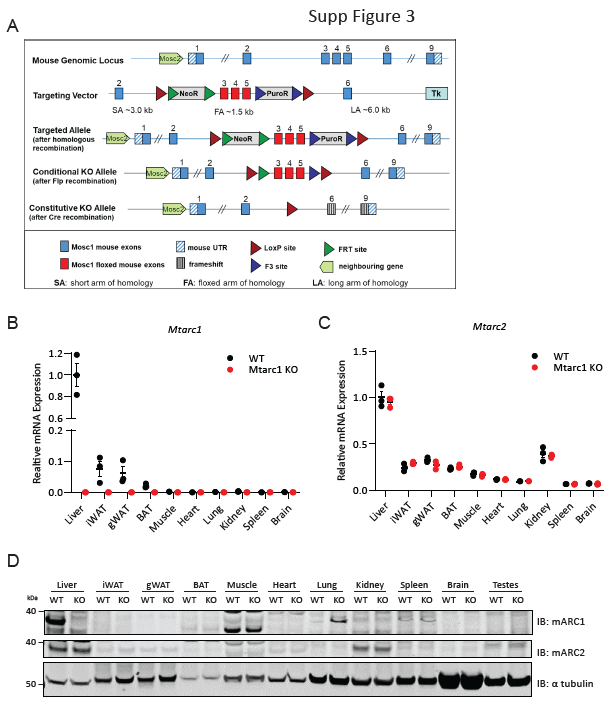
**

**Supplemental Figure 3: Generation of *Mtarc1* knockout mice**. (A) Schematic representation of engineering strategy to generate *Mtarc1* (*Mosc1*) knockout mice. (B) Expression of *Mtarc1* mRNA across various tissues in WT and *Mtarc1* KO mice n=3. (C) Expression of *Mtarc2* mRNA across various tissues in WT and Mtarc1 KO mice n=3. (D) Protein levels of mARC1 across various tissues in WT and KO mice.

**
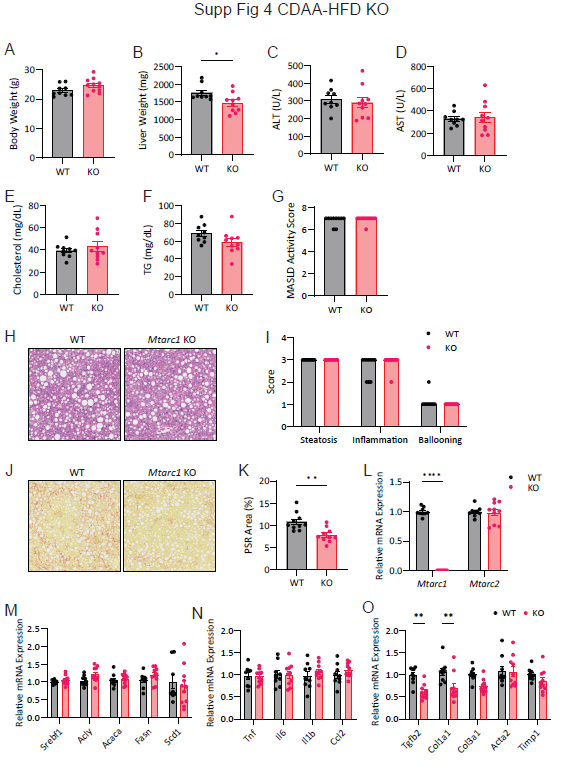
**

**Supplemental Figure 4: *Mtarc1* gene deletion results in decreased liver fibrosis in male mice fed choline deficient, amino acid-defined high fat diet**. Male WT and *Mtarc1* KO mice were fed a CDAA-HFD for 12 weeks. (A) Terminal body weight in WT and *Mtarc1* KO mice. (B) Terminal liver weight in WT and Mtarc1 KO mice. Plasma levels of (C) ALT, (D) AST, (E) cholesterol, and (F) triglycerides. (G) Pathologist-evaluated MASLD activity score. (H) Representative H&E images of WT and *Mtarc1* KO livers. (I) Pathologist-evaluated steatosis, inflammation, and ballooning scores. (J) Representative PSR stained section of WT and *Mtarc1* KO livers. (K) Quantification of PSR stained area. (L) *Mtarc1* and *Mtarc2* mRNA expression in the liver of WT and *Mtarc1* KO mice. (M) Lipogenic, (N) inflammatory, and (O) fibrosis gene expression. Data is presented as mean +/- SEM. n=9-10 *P<0.05, **P<0.01, ***P<0.001, ****P<0.0001.

**
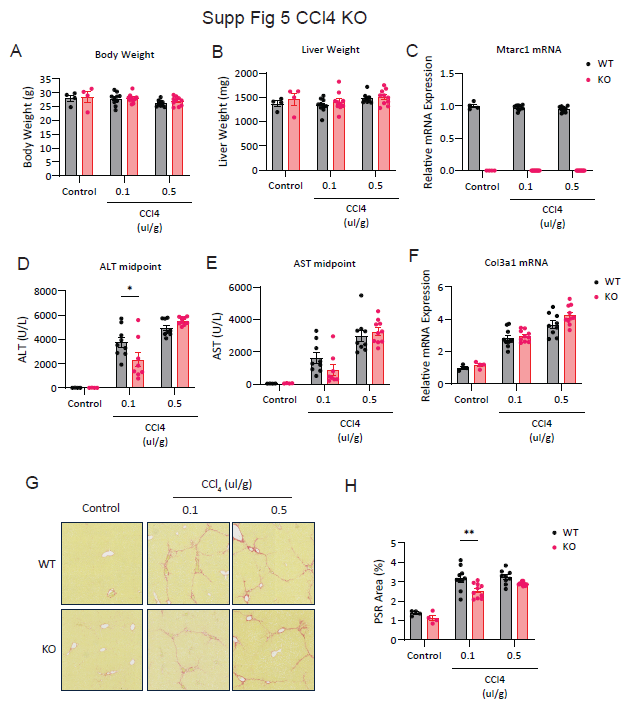
**

**Supplemental Figure 5: *Mtarc1* gene deletion results in decreased liver fibrosis in male mice fed after CCl_4-_induced liver injury.** Male WT and *Mtarc1* KO mice were challenged with hepatotoxin CCl_4_ (0.1 or 0.5 ugl/g) or olive oil control for 4 weeks. (A) Terminal body weight, (B) liver weight and (C) *Mtarc1* gene expression. (D) ALT and (E) AST levels 24 hours after CCl_4_ injection at the two-week timepoint. (F) mRNA expression of Col3a1 in the liver. (G) Representative PSR stained liver sections. (H) Quantification of PSR stained liver area. Data is presented as mean +/- SEM. n=4-8 *P<0.05, **P<0.01, ***P<0.001, ****P<0.0001.

**
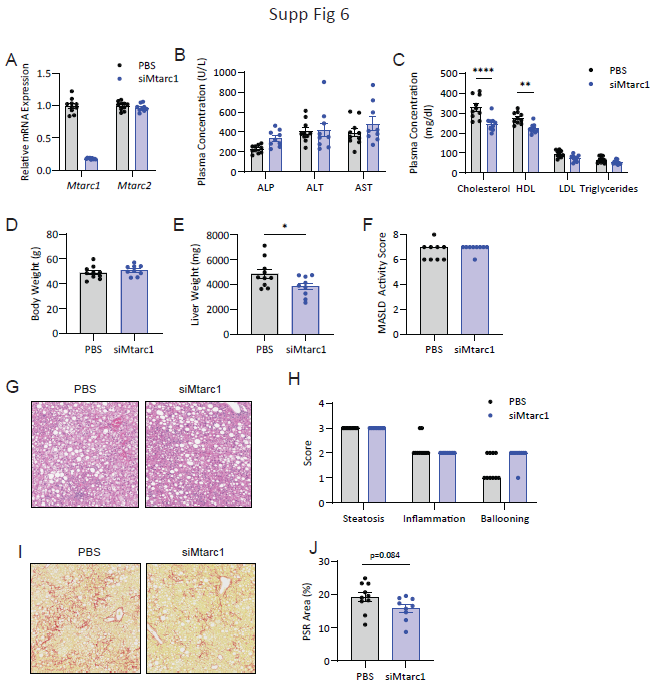
**

**Supplemental Figure 6: Hepatocyte-specific *Mtarc1* knockdown does not reduce liver fibrosis in male mice fed GAN diet after 32 weeks on diet.** Male C57BL/6J mice were fed GAN diet for 24 weeks to induce MASH and liver fibrosis and then treated with GalNAc-si*Mtarc1* or PBS Q2W for an additional 8 weeks. (A) mRNA expression of *Mtarc1* and *Mtarc2* in liver. (B) Plasma liver enzyme levels and (C) plasma lipid levels at the 32-week timepoint. (D) Body weight and (E) liver weight after 8 weeks of siRNA treatment and 32 weeks on diet. (F) Pathologist-evaluated MASLD activity score. (G) Representative H&E images of liver from PBS and siMtarc1 treated mice. (H) Pathologist-evaluated steatosis, inflammation, and ballooning scores. (I) Representative Picrosirius red (PSR) stained images of liver PBS and si*Mtarc1* treated mice. (J) Quantification of the PSR stained area within the whole liver lobe. Data is presented as mean +/- SEM. n=9-10 *P<0.05, **P<0.01, ***P<0.001, ****P<0.0001.

**
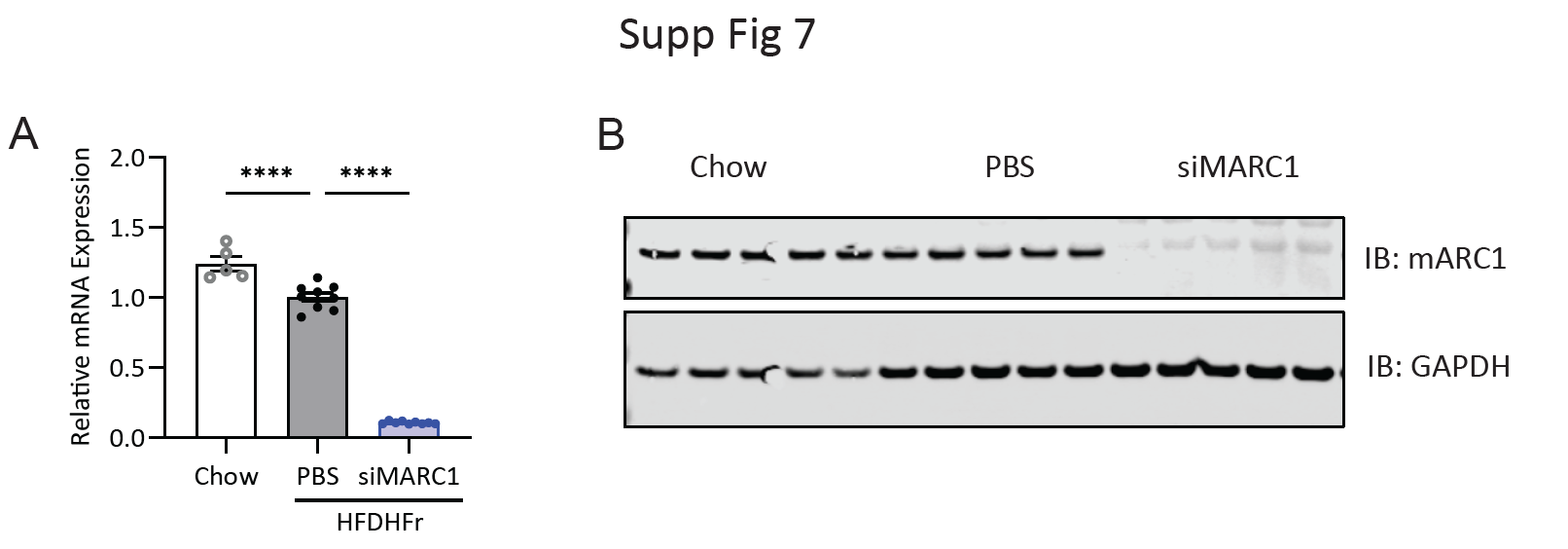
**

**Supplemental Figure 7: mARC1 is knocked down at the mRNA and protein level in livers of mice treated with GalNAc conjugated *Mtarc1* siRNA.** Male C57BL/6J mice were fed a high fat diet supplemented with fructose water for 12 weeks to induce MASH and liver fibrosis and then treated with GalNAc-si*Mtarc1* or PBS Q2W for an additional 8 weeks. Age-matched mice fed a normal chow diet were used as control. (A) Liver mRNA and (B) protein levels of *Mtarc1* at the 20-week timepoint.

**
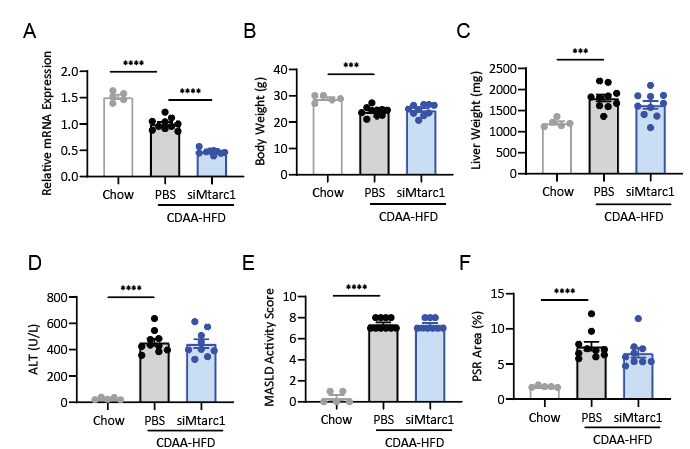
**

**Supplemental Figure 8: Hepatocyte-specific *Mtarc1* knockdown does not reduce liver fibrosis in male mice fed a CDAA-HFD.** Male C57BL/6J mice were fed CDAA-HFD diet for 4 weeks to induce MASH and liver fibrosis and then treated with GalNAc-si*Mtarc1* or PBS Q2W for an additional 4 weeks. (A) mRNA expression of *Mtarc1* in liver. (B) Body and (C) liver weight in mice at the 8-week timepoint. (D) Plasma liver enzyme ALT levels at the 8 week timepoint. (E) Pathologist-evaluated MASLD activity score and (F) quantification of the PSR stained area within the whole liver lobe at the 8 week timepoint. Data is presented as mean +/- SEM. n=5-10 *P<0.05, **P<0.01, ***P<0.001, ****P<0.0001.

**
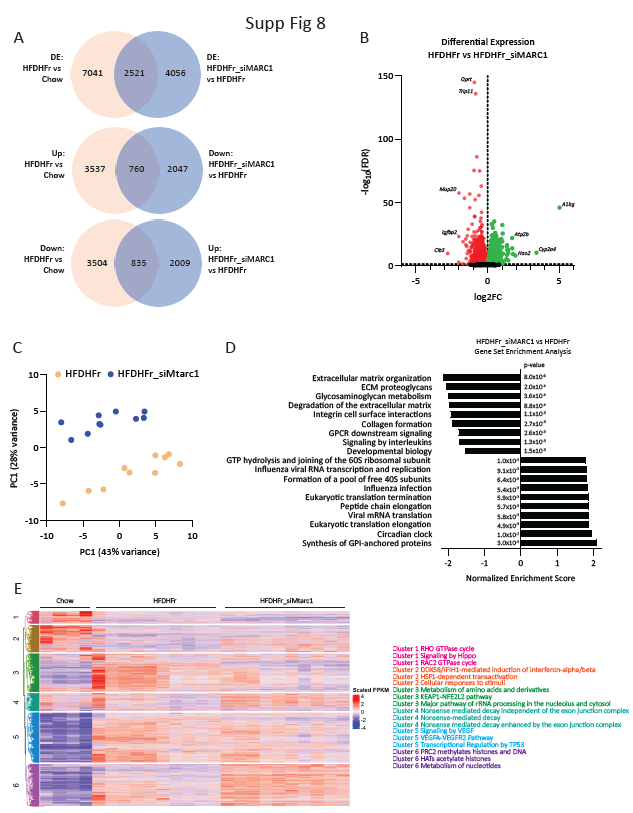
**

**Supplemental Figure 9: Liver RNA-seq profiling of hepatocyte-specific *Mtarc1* knockdown on liver gene expression in male mice fed a high fat diet supplemented with fructose water** (A) Visualization of number of differentially expressed genes in control mice fed a high fat diet with fructose water (HFDHFr) compared to mice fed a chow diet and overlap with differentially expressed genes in si*Mtarc1* treated mice compared with control HFDHFr mice. (B) Volcano plot visualization of differential gene expression in si*Mtarc1* treated mice compared with control HFDHFr mice with highly up or downregulated genes denoted. (C) Principal component analysis comparing si*Mtarc1* treated mice compared to control HFDHFr mice. (D) Gene set enrichment analysis visualization of normalized enrichment scores and p-values. (E) Clustered heat map and scaled FPKM of pathway analyses.

**
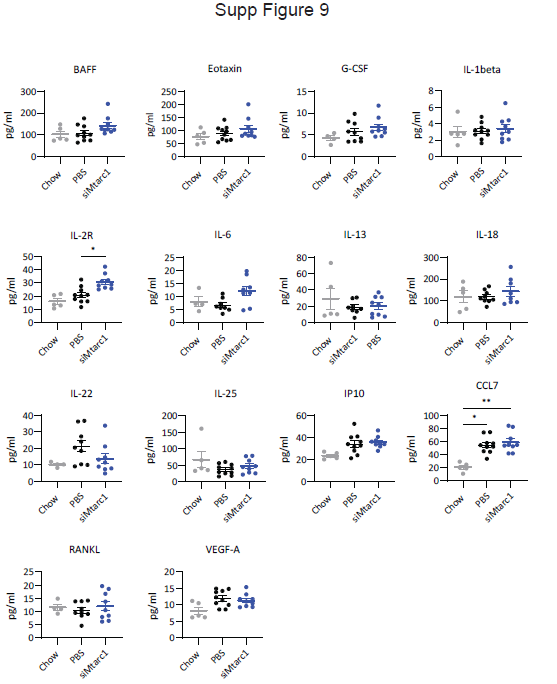
**

**Supplemental Figure 10: Plasma levels of immune markers in mice treated with Mtarc1 siRNA.** Male C57BL/6J mice were fed a high fat diet supplemented with fructose water for 12 weeks to induce MASH and liver fibrosis and then treated with GalNAc-si*Mtarc1* or PBS Q2W for an additional 8 weeks. Blood was collected at the end of the study and an immune marker panel of cytokines, chemokines and growth factors was assessed. Data is presented as mean +/- SEM. n=5-10 *P<0.05, **P<0.01.

**Supplemental Table 1: List of antibodies**

| **Antibody** | **Supplier** | **Catalog** |
| --- | --- | --- |
| MOSC1 | Novus Biologicals | NBP1-82122 |
| MARC2 | Protein Tech | 24782-1-AP |
| GFP | Cell Signaling Technology | 2555 |
| GAPDH | Cell Signaling Technology | 97166 |
| Alpha tubulin | Cell Signaling Technology | 3873 |

**Supplemental Table 2: List of Taqman Probes**

| **Gene** | **Probe** |
| --- | --- |
| Tbp | Mm01277042_m1 |
| Srebf1 | Mm00550338_m1 |
| Acly | Mm01302282_m1 |
| Acaca | Mm01304285_m1 |
| Fasn | Mm00662319_m1 |
| Scd1 | Mm00772290_m1 |
| Tnf | Mm00443258_m1 |
| Il6 | Mm00446190_m1 |
| Il1b | Mm01336189_m1 |
| Ccl2 | Mm00441242_m1 |
| Col1a1 | Mm00801666_g1 |
| Col3a1 | Mm00802300_m1 |
| Acta2 | Mm01546133_m1 |
| Timp1 | Mm01341361_m1 |
| TBP | Hs00427620_m1 |
| MARC1 | Hs00224227_m1 |
| MARC2 | Hs00215486_m1 |
| DDIT3 | Hs99999172_m1 |
| PLIN2 | Hs00605340_m1 |
| NRF1 | Hs00602161_m1 |
| NFE2L2 | Hs00975961_g1 |
| SOD1 | Hs00533490_m1 |
| PPARA | Hs00947536_m1 |
| CPT1A | Hs00912671_m1 |
| SCD | Hs01682761_m1 |
| PPARGC1A | Hs00173304_m1 |

**References**

1. Tremblay F, Mcininch JD, Inventors. Compositions and Methods for Inhibiting MARC1 Gene Expression. US patent WO2021237097A12021.

2. Liu F, Goh GB, Tiniakos D, et al. qFIBS: An Automated Technique for Quantitative Evaluation of Fibrosis, Inflammation, Ballooning, and Steatosis in Patients With Nonalcoholic Steatohepatitis. *Hepatology.* 2020;71(6):1953-1966.

3. Naoumov NV, Brees D, Loeffler J, et al. Digital pathology with artificial intelligence analyses provides greater insights into treatment-induced fibrosis regression in NASH. *J Hepatol.* 2022;77(5):1399-1409.

4. Abousamra S, Belinsky D, Van Arnam J, et al. Multi-Class Cell Detection Using Spatial Context Representation. *Proc IEEE Int Conf Comput Vis.* 2021;2021:3985-3994.

5. Schmidt U WM, Broaddus C, Myers G. Cell detection with star-convex polygons. *Medical Image Computing and Computer Assisted Intervention.* 2018:265-273.

6. Weigert M SU, Haase R, Sugawara K, Myers G. Star-convex polyhedra for 3D object detection and segmentation in microscopy. *Proceedings of the IEEE CVF winter conference on applications of computer vision* 2020:3666-3673.

7. Macenko M, Niethammer M, Marron JS, et al. A method for normalizing histology slides for quantitative analysis. Paper presented at: IEEE international symposium on biomedical imaging: from nano to macro2009.

8. Ronneberger O, Fischer P, Brox T. U-net: Convolutional networks for biomedical image segmentation. Paper presented at: Medical image computing and computer-assisted intervention–MICCAI 2015: 18th international conference2015; Munich, Germany.

9. He K, Zhang X, Ren S, Sun J. Identity mappings in deep residual networks. Paper presented at: Computer Vision–ECCV 2016: 14th European Conference2016; Amsterdam, The Netherlands.

10. Ilse M, Tomczak J, Welling M. Attention-based deep multiple instance learning. Paper presented at: International conference on machine learning2018.

11. Love MI, Huber W, Anders S. Moderated estimation of fold change and dispersion for RNA-seq data with DESeq2. *Genome Biol.* 2014;15(12):550.

12. Gu Z. Complex heatmap visualization. *Imeta.* 2022;1(3):e43.

13. Yu G, Wang LG, Han Y, He QY. clusterProfiler: an R package for comparing biological themes among gene clusters. *OMICS.* 2012;16(5):284-287.

14. Korotkevich G, Sukhov V, Sergushichev A. [Pre-print] Fast gene set enrichment analysis. *bioRxiv* 2019.

15. Surma MA, Herzog R, Vasilj A, et al. An automated shotgun lipidomics platform for high throughput, comprehensive, and quantitative analysis of blood plasma intact lipids. *Eur J Lipid Sci Technol.* 2015;117(10):1540-1549.

16. Mohamed A, Molendijk J, Hill MM. lipidr: A Software Tool for Data Mining and Analysis of Lipidomics Datasets. *J Proteome Res.* 2020;19(7):2890-2897.

17. Ritchie ME, Phipson B, Wu D, et al. limma powers differential expression analyses for RNA-sequencing and microarray studies. *Nucleic Acids Res.* 2015;43(7):e47.
